# Behavioral Classification of Sequential Neural Activity Using Time Varying Recurrent Neural Networks

**DOI:** 10.1109/TNSRE.2025.3586175

**Published:** 2025

**Authors:** Yongxu Zhang, Catalin Mitelut, David J. Arpin, David Vaillancourt, Timothy Murphy, Shreya Saxena

**Affiliations:** Department of Biomedical Engineering, Yale University, New Haven, CT 06520 USA; Department of Biology, New York University, New York, NY 10003 USA; Department of Applied Physiology and Kinesiology, University of Florida, Gainesville, FL 32611 USA; Department of Applied Physiology and Kinesiology, University of Florida, Gainesville, FL 32611 USA; Department of Psychiatry, Kinsmen Laboratory of Neurological Research, The University of British Columbia, Vancouver, BC V6T 2A1, Canada; Department of Biomedical Engineering, Yale University, New Haven, CT 06520 USA

**Keywords:** Sequential classification, neural activity, widefield calcium imaging, fMRI, recurrent neural networks, gradient vanishing/exploding, early classification, contribution of brain regions

## Abstract

Shifts in data distribution across time can strongly affect early classification of time-series data. When decoding behavior from neural activity, early detection of behavior may help in devising corrective neural stimulation before the onset of behavior. Recurrent neural networks are common models for sequence data. However, standard recurrent neural networks are not able to handle data with temporal distributional shifts to guarantee robust classification across time. To enable the network to utilize all temporal features of the neural input data, and to enhance the memory of recurrent neural networks, this paper proposes a novel approach: recurrent neural networks with time-varying weights, here termed Time-varying recurrent neural networks. These models are able to not only predict the class of the time-sequence correctly, but also lead to accurate classification earlier in the sequence than standard recurrent neural networks, while also stabilizing gradient dynamics. This paper focuses on early sequential classification of spatially distributed neural activity across time using Time-varying recurrent neural networks applied to a variety of neural data from mice and humans, as subjects perform motor tasks. Time-varying recurrent neural networks detect self-initiated lever-pull behavior up to 6 seconds before behavior onset—3 seconds earlier than standard recurrent neural networks. Finally, this paper explored the contribution of different brain regions on behavior classification using SHapley Additive explanation value, and found that the somatosensory and premotor regions play a large role in behavioral classification.

## I. INTRODUCTION

Robust classification of behavior from multi-regional sequential neural data has garnered increasing attention in recent years [1], [2], [3]. Temporal neural activity can be classified sequentially in time, which has the potential for early detection of behavior. However, achieving both accurate classification of the entire sequence and maintaining a dynamic estimate of classification reliability presents a challenge, particularly in the presence of temporal distributional shifts in the data. This study investigates accurate classification of behavior from neural time-series as early and reliably as possible. Specifically, this paper focuses on predicting behavior before its occurrence, while ensuring robust classification over time, in the face of temporal variations in the data [4].

**Fig. 1.**
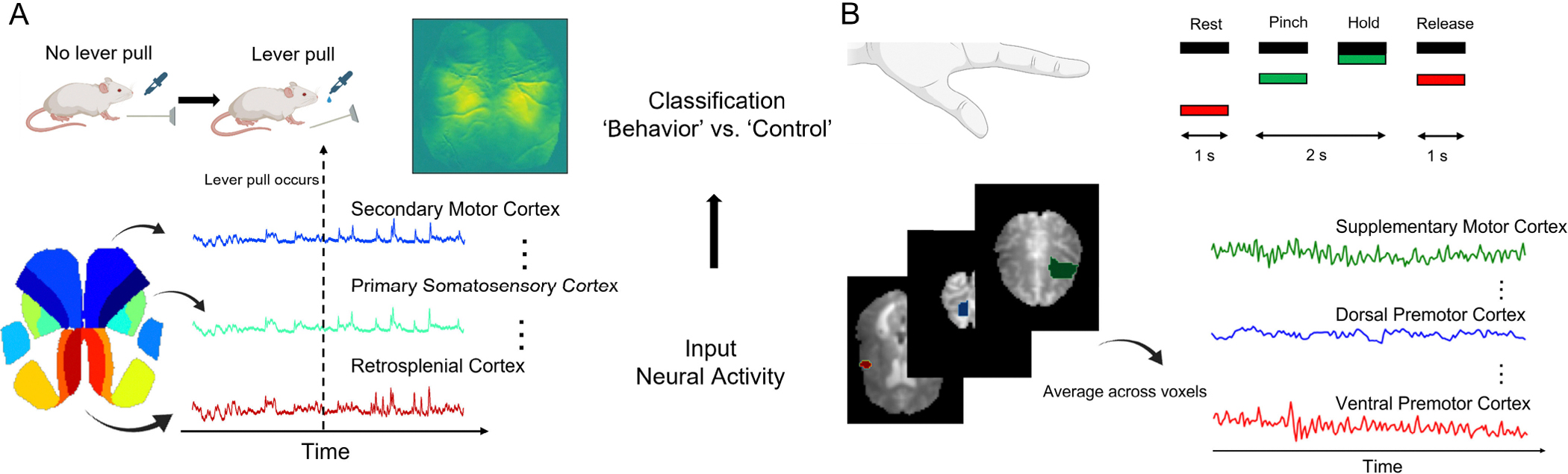
(A) In the WFCI dataset, mice were trained to pull a lever for water reward, while WFCI activity was recorded from multiple regions. (B) Neural activity of healthy and PD human subjects in a grip force task was recorded using fMRI.

Recurrent neural networks (RNNs) are designed for time-series data: they take in sequential inputs and, if optimized for classification, predict the class of the sequence using recurrent hidden states that are able to retain a memory of previous inputs. However, conventional RNNs are inherently static, which may limit their performance on long time-series data with evolving statistical properties. Instead, they excel at accurately classifying temporal data primarily at the end of the sequence. To aid the network utilization of all temporal features of the input and to enhance the memory of an RNN, this paper proposes a novel approach: RNNs with time-varying weights, termed Time-varying RNNs (TV-RNNs). These models are able to not only predict the class of the sequence correctly, but also lead to accurate classification earlier in the sequence than standard RNNs. This work, with TV-RNNs, focuses on the early sequential classification of brain-wide neural activity across time, as subjects perform a motor task (Fig 1A). Three different datasets are used: (1) simulated data with chirp signals to simulate distributional shifts in the data, (2) **widefield calcium imaging (WFCI)** records the neural activity across mouse dorsal cortex while subjects perform a ‘lever pull’ task, and (4) **functional magnetic resonance imaging (fMRI)** that records human whole-brain neural activity while patients with Parkinson’s Disease (PD) and healthy controls perform a ‘grip force’ task.

The main contributions of this work are summarized as follows:

- This study proposes Time-varying RNNs, a novel architecture that adapts its recurrent weights over time to better capture temporal dynamics in sequential data. These models are able to not only predict the class of the sequence correctly, but also lead to accurate classification earlier in the sequence than standard RNNs.
- Time-varying RNNs improve training stability, mitigating issues of vanishing or exploding gradients often encountered in standard RNNs.
- The underlying classification mechanisms of Time-varying RNNs are interpretable, and reveal how their temporal flexibility contributes to decision-making.
- Through the use of SHapley Additive explanation (SHAP) values, this study finds that the effect of different regions on behavioral decoding is varied; the somatosensory and premotor regions play a large role in behavioral classification across both mice and humans.

The remainder of this paper is organized as follows: Section II reviews related work, Section III details the proposed methods and experiments, Section IV presents the experimental results, and Section V concludes with a discussion of the findings, limitations and future directions.

## II. RELATED WORK

### A. Sequential Classification

Previous work has decoded behavior by dividing data into windows and applying independent classifiers to each, such as Support Vector Machines (SVMs) predicting decisions seconds before awareness [1], [5], [6]. However, the temporal information hidden in the time series data is not adequately utilized in these models because each classifier is independent. RNNs address this by modeling neural data sequentially, capturing temporal dynamics more effectively. Classification of sequence data has attracted extensive attention and can be applied in many areas, e.g., genomic analysis, information retrieval, and health informatics [7]. Information about the data is stored across the sequence; this work considers neural data in which the features for predicting the behavior are not only distributed sequentially in time but also across different regions of the brain. RNNs continue to show outstanding performance in sequence learning tasks such as language modeling [8], [9]. Moreover, by using and storing information across the sequence, RNNs are able to convert their representations across time to adapt to the task, and thus, they perform well in classifying sequential data [10]. RNNs are also widely used for sequential data analysis in other research domains, such as economics [11], web services [12], cloud computing [13], space weather prediction [14], and biomedical signal analysis [15]. Furthermore, predicting the class of a time series as early as possible is crucial for enabling timely interventions. In Xing et al., the authors explored the minimal prediction length for neural networks to classify time-series data accurately [16]. In Mori et al., the authors optimized the early index and accuracy of a network at the same time [4]. Here, a time-varying approach is used to perform early and sequential classification of behavior using neural data. Transformers have shown effectiveness in sequential classification tasks [17], [18], however, their original architecture is not well-suited for temporal classification, as the self-attention models typically rely on the entire input sequence during decoding, including future time points, which is incompatible with real-time or causal classification tasks. Consequently, to ensure that the model makes predictions at time t without accessing future information, causal mask embedding is performed in this paper.

### B. Temporal Distribution Shifts

Many algorithms have been proposed to address distributional shifts of data to improve the model’s performance on classification. Typically, the methods based on classical supervised learning use sliding window methods to overcome the effect of distribution changes on the model’s performance [5], [19]. However, sliding window methods need a large number of classifiers and these classifiers are usually not connected to each other, therefore, the model may not get continuous information which is important in uncovering the computational mechanisms. Additionally, this challenge is also addressed using feature adaptation via transformations of future representations [20], [21]. Yet, in most cases, feature adaptation is applied to sequential classification and the models may not be able to classify at each time point due to the potential change of feature representation across time. Consequently, RNNs are chosen as the base model because of their ability to process continuous information and generate outputs at each time point. Despite being implementable based on the universal approximation theory, very large models lack interpretability and fail to capture the essential temporal distribution shifts in data. Therefore, relatively low-dimensional models are chosen in this study. Furthermore, RNNs are capable of uncovering underlying computational mechanisms, which potentially offers insights into the neural mechanisms underlying behavior [22], [23].

### C. Time Varying Models

Models with time varying parameters are efficient in dealing with temporal tasks, they have unique parameters to utilize specific information at different times. For instance, switching linear dynamical systems (SLDS) and recurrent SLDS are designed to parse data sequences into coherent discrete units which help to capture distinct dynamics in different time periods of time-series data [24]. Moreover, time varying regression models have shown their utility in a range of applications such as economics [25], [26] and disease analysis [27]. Classification can also be improved by applying different parameters temporally, e.g., Yang et al., used multiple convolutional neural networks in parallel across time to classify time varying signals [28], and Wang et al., show that time varying parameters outperform common machine learning approaches in the classification of EEG signals [29]. However, these methods lack connections between different parts of the models: temporal information that may be crucial for classification is not transmitted across time. On the contrary, the proposed TV-RNNs here have explicit hidden states storing and transmitting temporal information across the entire sequence.

### D. Recurrent Neural Networks

With the rapid development of artificial intelligence, various powerful models have emerged that have outstanding performance in temporal classification; examples include Transformer models as well as temporal Convolutional Neural Networks (CNNs) [30], [31], [32]. According to the Universal Approximation Theorem, highly complex models or large models can achieve effective temporal classification. However, their interpretability is often limited. This paper focuses not only on achieving efficient temporal classification but also on enhancing interpretability on the relationship between behavior and changes in neural dynamics. Compared with more complex models, RNNs offer a distinct advantage, particularly in the context of interpretability and dynamics. In contrast to Transformers, which rely on self-attention mechanisms and extensive parametrization, RNNs explicitly model temporal dependencies through recurrent connections, making them well-suited for capturing the underlying dynamics of sequential data. This inherent structure allows RNNs to provide more direct insights into temporal evolution, enabling researchers to analyze state transitions and neural representations more effectively [33]. This work also demonstrates that classification mechanisms exhibit evidence accumulation when using TV-RNNs. Additionally, the temporal evolution of weights aligns with shifts in the data distribution.

## III. METHODS

This section provides details of proposed model TV-RNNs and introduces the metrics used to quantify the classification performance of these models and the importance of different brain regions. In addition, the experimental details of the datasets are also shown in this section.

### A. Standard Recurrent Neural Networks

**Fig. 2.**
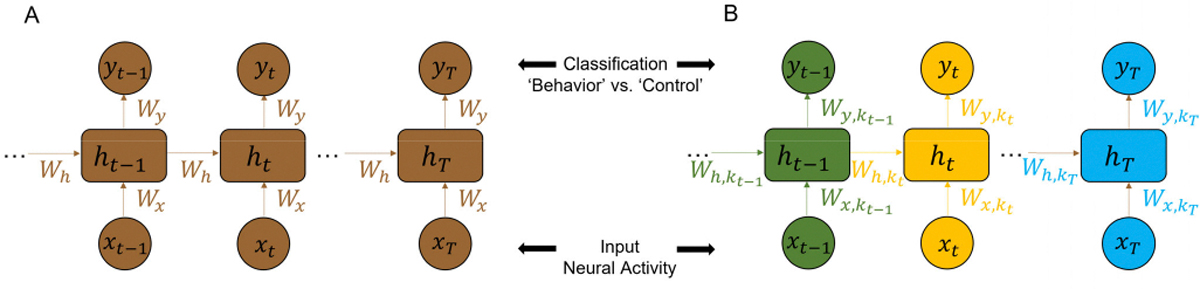
(A) Standard RNNs and (B) Time-Varying RNNs (TV-RNNs) used for behavioral classification of neural activity from different brain regions.

A classification model is built with time-series neural data $x\in R^{R\times T}$ from R different brain regions and T time points as the input, with the outputs as the different classes of behavior. Here, a hidden recurrent layer is implemented with the *tanh* activation function, and a dense layer at the output with the *sigmoid* activation function σ to predict the binary class. Following are the equations of the RNN network.

(1) $$h_{t}=tanh\left(W_{h}h_{t-1}+W_{x}x_{t}+b_{h}\right)\;\forall t\in [1,T]$$

(2) $$y_{t}=\sigma \left(W_{y}h_{t}+b_{y}\right)\;\forall t\in [1,T]$$

(3) $$c=\left\{\begin{array}{ll} 0, & if\;y_{t}<0.5\\ 1, & otherwise \end{array}\right.$$

where $x_{t}\in R^{R\times 1}$ is the neural data from all R brain regions at time point $t,\;h_{t}\in R^{N\times 1}$ is the value for the N hidden units at time point $t,\;W_{x}\in R^{N\times R}$ is the input weight matrix, $W_{h}\in R^{N\times N}$ contains the recurrent weights for the hidden layer, and $W_{y}\in R^{1\times N}$ represents the output weight matrix. $y_{t}$ is the output of dense layer. Figure 2A shows the specific structure of the unfolded standard RNNs. Backpropagation-thru-time (BPTT) is used to train the RNNs. Also, two commonly-used loss functions are applied to train the standard RNNs: (a) the loss at the last output of RNNs $\left(y_{T}\right)$ in order to focus on the prediction of the entire sequence (termed ‘S1’ here); and (b) the loss at all time steps of RNNs sequence $\left(\sum_{t}y_{t}\right)$, where the model focuses on not only the prediction at the end of the sequence, but also on the aggregate performance of the RNNs (termed ‘S2’ here). Note that only one set of weights need optimizing in both cases. The pseudo-codes are shown in Supplementary in Algorithms 1 and 2 respectively.

### B. Time-Varying Recurrent Neural Networks

In order to capture the specific temporal features of the input, an RNN is designed with time-varying weights including input weights $W_{x}^{t}$, recurrent weights $W_{h}^{t}$, output weights $W_{y}^{t}$, and biases $b_{h}^{t}$, $b_{y}^{t}$.

(4) $$h_{t}=tanh\left(W_{h}^{t}h_{t-1}+W_{x}^{t}x_{t}+b_{h}^{t}\right)\;\forall t\in [1,T]$$

(5) $$y_{t}=\sigma \left(W_{y}^{t}h_{t}+b_{y}^{t}\right)\;\forall t\in [1,T]$$

(6) $$W_{x,h,y}^{t}=W_{x,h,y}^{k}\;\forall t\in [(k-1)w,kw],k\in \left[1,\frac{T}{w}\right]$$

(7) $$c=\left\{\begin{array}{ll} 0, & if\;y_{t}<0.5\\ 1, & otherwise \end{array}\right.$$

where w is the window size of RNNs. Specifically, the inputs in each time window w are fed into RNNs with one set of input weights $W_{x}^{t}$, recurrent weights $W_{h}^{t}$, output weights $W_{y}^{t}$, and bias $b_{h}^{t},\;b_{y}^{t}$. Thus, for the entire sequence of inputs, $\frac{T}{w}$ sets of weights are used. Fig 2B shows the specific structure of the unfolded TV-RNNs. In TV-RNNs, multiple sets of weights need to be trained. The optimization of all the TV-RNN weights is performed simultaneously (end-to-end) with the same BPTT, i.e., in each batch. The pseudo-code of training TV-RNNs is shown in Algorithm 3 in Supplementary. In all cases of standard RNNs and TV-RNNs, the number of hidden units is 64, and all networks are trained for 1000 epochs using Adam at a learning rate of 0.0001. These hyperparameters were determined using cross-validation on a sample session of the dataset. Pytorch is used to train all models. All tasks are performed on HiPerGator Computational Supercomputer at the University of Florida, with NVIDIA A100 GPUs. The code is available online https://github.com/saxenalab-neuro/TV_RNN.

### C. Transformer

The Transformer architecture, originally developed for natural language processing, has shown strong performance in various sequence modeling tasks due to its ability to capture long-range dependencies using self-attention [30]. Here, the performance of TV-RNNs is quantitatively compared with Transformer models. However, standard Transformers typically rely on the entire input sequence during decoding, including future time points, which is incompatible with real-time or causal temporal classification tasks. To address this issue, this work uses a causal Transformer that restricts attention to past and current time steps only, ensuring that the model makes predictions at time t without accessing future information – a critical requirement for sequential decision-making in real-world scenarios. We also evaluated a standard (non-causal) Transformer but observed no significant performance improvement over the causal version.

### D. Accuracy Quantification

#### 1) Temporal Accuracy:

In order to describe the performance of temporal decoding and exploring early classification, temporal accuracy *Accuracy*_*t*_, which depicts the classification accuracy at each time point t, is used in this work, and 5-fold cross-validation is applied to all of the experiments. In the following, the data are split into $\frac{1}{5}$ of test set in each fold, during training, the rest $\frac{4}{5}$ set is split into training set and validation set with validation rate of 0.2, the validation set is used to monitor over-fitting during training, and thus decide the hyperparameters, i.e., learning rate, training iterations, batch size, and the TV-RNN window size w. All results are reported on test data Accuracy here is defined as $\frac{1}{K}\sum_{k=1}^{K}\frac{TP(k)+TN(k)}{TP(k)+TN(k)+FP(k)+FN(k)}$, where K is the number of folds, TP(k) is true positives in the $k^{th}$ fold, TN is true negatives, FP is false positives, and FN is false negatives.

#### 2) Area Under Accuracy Curve (AUAC):

The area under the accuracy curve above chance level in different time windows quantifies the overall decoding ability of the classifier.

#### 3) Earliest Decoding Time:

This work aims to classify the sequence as early and as accurately as possible. In order to explore the ability of RNNs in early classification, a metric called *earliest decoding time*, is used to measure early classification. This is the earliest time point after which the models obtain consistent and significant decoding till behavior onset. Significance was determined using a one-tailed t-test at a significance level of p<0.05 (after multiple hypothesis correction using the Benjamini-Hochberg procedure [34]). Therefore, the earliest decoding time represents the earliest time after which the behavior can be reliably decoded.

### E. Importance of Different Brain Regions

The important features for decoding are stored in both the time domain and spatial domain of the data Here, the spatial domain encompasses the different recorded brain regions in the WFCI or fMRI modalities. In previous work, the occlusion method was explored to quantify the importance of each region in both the time and brain region domain [6]. In this work, SHapley Additive exPlanation (SHAP) value is employed, it is able to overcome the limitations of occlusion, because it considers the complete effect of a region on classification. SHAP value is firstly introduced by Lundberg and Lee in [35]. It interprets the effect of a given feature on the output of the model explained by computing Shapley values from coalitional game theory [36]. In binary classification, a positive value means the contribution of a given feature to predict a positive class, meanwhile, a negative value reflects the contribution to predicting a negative class. Therefore, the absolute SHAP value is used which is able to easily represent the contribution of the feature towards binary classification. SHAP and importance are defined as follows:

(8) $$\phi_{i}=\sum_{S\subseteq F\backslash \{i\}}\frac{|S|!(|F|\;-\;|S|\;-\;1)!}{|F|!}\left[f_{S\cup \{i\}}\left(x_{S\cup \{i\}}\right)-f_{S}\left(x_{S}\right)\right]$$

(9) $$I_{i}=\frac{1}{N}\sum_{n=1}^{N}\left|\phi_{n,i}\right|$$

where $\phi_{i}$ is the SHAP value of feature i,F and S mean all the features and subsets of all the features, here, features are in both time domain and brain region domain. Additionally, f represents classifiers, i.e., standard RNNs and TV-RNNs, $I_{i}$ indicates the importance of feature i which is an average of SHAP value of this feature across all trials. In this work, GradientExplainer is employed, which is based on integrated gradient values to approximate the SHAP values. The integrated gradients are defined by Sundararajan et al., in [37] as:

(10) $$IG_{i}\left(x\right)\;:\;:=\left(x_{i}-x_{i}^{\prime }\right)\times \int_{\alpha }^{1}\frac{\partial F\left(x^{\prime }\;+\;\alpha \;\times \;\left(x\;-\;x^{\prime }\right)\right)}{\partial x_{i}}d\alpha$$

where x is the input used to explain the model, here the temporal neural activity in the test set, and $x^{\prime }$ represents the baseline input. The integrated gradients are crucial to approximate the SHAP value.

(11) $$\sum_{i}^{n}IG_{i}(x)=F(x)-F\left(x^{\prime }\right)$$

In this case, $F\left(x^{\prime }\right)$ is around 0.5 since the *sigmoid* activation function is used in the output layer for both standard and TV-RNNs. Therefore, the difference between the outputs of subset with explained features and the subset without explained features in Eq. 8 is able to be approximated by integrated gradients.

### F. Experimental Methods

#### 1) Simulated Dataset:

In order to validate TV-RNNs where the ground truth is known, a simulated dataset is generated with very clear features. These features simulate the properties in neural data that can be leveraged for classification. The simulated behavior data consists of 10 chirp signals with 300 time points in each trial. Each of 10 signals is multiplied by a distinct coefficient selected within a range of *(*1, 4*)*, and the amplitude linearly increases across time points in each signal. Gaussian noise is then added, and the simulation contains 2000 trials. The simulated *‘control’* signals are shuffled *‘behavior’* signals across time.

#### 2) Widefield Calcium Imaging (WFCI) Dataset:

Widefield experiments record large-scale neural activity from the mouse dorsal cortex through WFCI. The widefield neural activity is analyzed while mice engage in a task. In the experiments, head-fixed water-deprived mice were trained to pull a lever and hold it at an angle (for *>* 100ms) in order to receive a water supplement. Rewarded lever pulls were identified online (using a lever analog signal), and a minimum 3 seconds lockout window was used to make sure the mice cannot get rewarded twice for less than 3 seconds. Mouse protocols were approved by the University of British Columbia Animal Care Committee and followed the Canadian Council on Animal Care and use guidelines (protocols A13-0336 and A14-0266). Widefield calcium imaging was recorded from the mouse dorsal cortex and pre-processed as previously described [38]. The details of pre-processing were explained previously in [5]. Additionally, the entire dataset is publicly available at https://doi.org/10.5061/dryad.ttdz08m0z. To ensure reproducibility, the dataset in this paper will also be released with the code upon publication at the following link: https://github.com/saxenalab-neuro/TV_RNN. This work identifies the *‘behavior’* trials as trials that were tracked in real time to provide water reward, with the trial centered around the initiation of the lever pull behavior. As control trials, the time of the lever pull behavior was randomized to fall anywhere except a ±3 s window around the lever pull behavior, and the same number of time points was selected for the *‘control’* trials as the *‘behavior’* trials. Thus, the *‘behavior’* trials have a clear behavior initiated at the middle of the trial, unlike the *‘control’* trials. In order to further eliminate the influence of multiple instances of lever pulls occurring during a *‘behavior’* trial, trials are manually selected such that only one instance of lever pull is located at the middle of each *‘behavior’* trial. The neural activity is sampled at 30 time points per second, and each trial in this dataset contains 1800 time points (60 seconds). The imaged neural activity is spatially aligned with the Allen mouse brain coordinate framework [39] using affine transformations, as previously performed in [40] and [41]. Then, localized semi-nonnegative matrix factorization (LocaNMF) [41] is applied on WFCI and 16 components are identified by LocaNMF, which form the input signals, with each input dimension from one brain region. This work focuses on the signals around the lever pull, i.e., from 10 seconds before lever pull to 0 second after lever pull, because *‘behavior’* trials and *‘control’* trials are easier to be classified during these periods [5], [6]. To increase trial counts, data is pooled across all sessions of each mouse. The average number of *‘behavior’* trials across all mice is 2620, and the number of *‘control’* trials is the same as *‘behavior’* trials. Additionally, the quantification of classification performance is shown by using per-session data in sessions with greater than 39 trials to maintain a large trial count.

#### 3) Functional Magnetic Resonance Imaging (fMRI) Dataset:

An fMRI force production paradigm was used to assess differences in brain activity between patients with Parkinson’s Disease and healthy age-matched controls. Participants were required to perform a grip force task which consists of pinching the force transducer for 2 seconds, then releasing for 1 second, with visual displays presented during the task. Participants were asked to rest for 30 seconds before they start a grip force task, then perform the task for 30 seconds. They repeat this alternating rest-task procedure 4 times. The experiment was approved by the University of Florida Institutional Review Board (IRB), protocol number 201600872. All participants provided informed consent. Details are included in [42]. In this work, this dataset is used to test the ability of TV-RNNs towards a large variety of neural data. The fMRI data is aligned to the Human Motor Area Template (HMAT) developed in [43], and the fMRI signals are averaged across voxels in each of 12 brain regions as shown in Fig 1B. The fMRI signal during subjects performing the grip force task is regarded as *‘behavior’*, with the rest procedure being *‘control’*, therefore, this is a binary classification task as well. Each trial has 12 time points representing 30 seconds in real time. In each group (patients or healthy control), RNNs classify all *‘behavior’* and *‘control’* trials, i.e., grip force versus rest. The classification performance between Parkinson’s Disease patients and healthy controls is also compared. The dataset contains 46 patients and 34 healthy controls, the number of *‘behavior’* trials in Parkinson’s Disease patients is 184, and the number of *‘behavior’* trials in healthy controls is 136.

## IV. RESULTS

The data distribution of the datasets is first analyzed, Behavioral classification is then performed using the proposed models and baseline models, demonstrating that TV-RNNs outperform standard RNNs. Next, the computational mechanisms of the TV-RNNs during classification is examined. Finally, the effect of different regions on the classification accuracy is investigated.

### A. Data Distribution

**Fig. 3.**
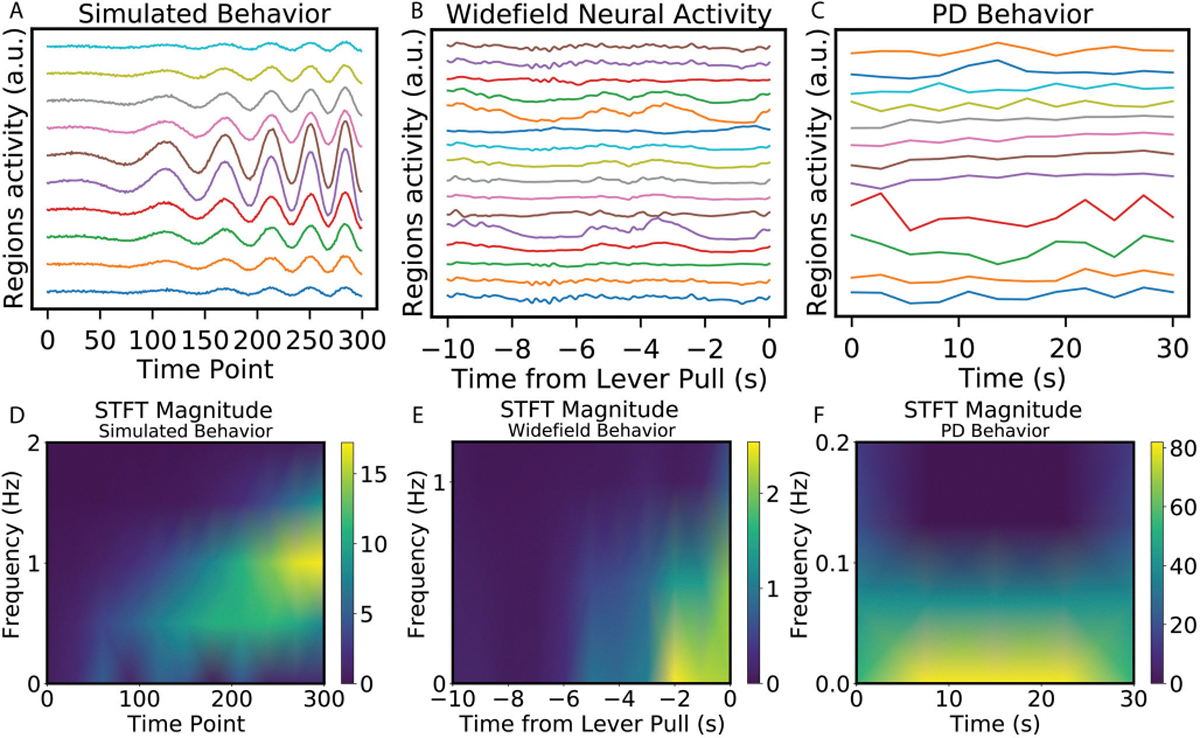
Plot of an example (A) simulated *‘behavior’* trial, (B) WFCI *‘behavior’* trial, (C) fMRI *‘behavior’* trial. Short Time Fourier Transform (STFT) magnitude of (D) simulated behavior signal, (E) WFCI dataset and (F) fMRI dataset.

Example trials of simulated behavior and simulated control signal are shown in Fig 3A. In order to visualize the shifts in data distribution, the STFT magnitude is shown in Fig 3B. This reveals the data shifting over time from low frequency and low amplitude to high frequency and high amplitude. The data distribution of the WFCI dataset (Fig 3C) and the fMRI dataset (Fig 3D–F) are also visualized. The STFT magnitude illustrate a shift of data distribution existing in the WFCI data at around 5 seconds before the lever pull. However, the data distribution of the fMRI data is relatively static: the data has similar frequency and magnitude across time.

### B. Classification Accuracy Using RNNs

#### 1) Simulated Dataset:

**Fig. 4.**
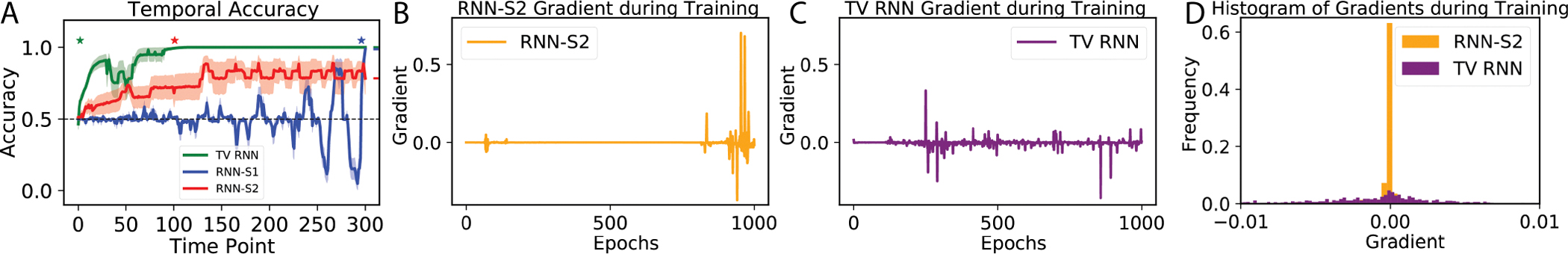
(A) Temporal classification accuracy curve of standard RNNs and TV-RNNs using simulated data. The stars on top represent the earliest decoding time for each model (see Methods), and the bars on the right side reflect the final classification accuracy of the sequence. Note that chance accuracy level is 0.5 for both datasets. Gradients of an example recurrent weight during training (first 1000 epochs) in (B) RNN-S2 and (C) TV-RNN show that TV-RNNs are able to reduce the probability of gradient vanishing and exploding (see Methods). All other recurrent weights have similar gradients plot (not shown). (D) Histogram of gradients during training shows that TV-RNN has less small gradients, i.e., gradient vanishing.

The temporal classification accuracy of a simulated dataset between standard RNNs and TV-RNNs is compared. A common strategy: BPTT with a binary cross-entropy loss using the output at the end of the sequence (RNN-S1) is first applied. Fig 4 (blue curve) shows that the temporal classification accuracy using this strategy only starts to increase above chance level after 150 time points. This trend matches the signal statistics in Fig 3D: after 150 time points, the simulated behavior signal has a higher frequency and magnitude. The alternative strategy to train Standard RNNs (RNN-S2), BPTT with the sum of the binary cross-entropy loss over time, leads to a low final accuracy (Fig 4, red curve). Consequently, a single set of weights in the standard RNNs does not seem to be able to guarantee early and accurate classification. On the other hand, TV-RNNs (Fig 4, green curve) can not only predict the class of the sequence early in the sequence, but maintain a high classification accuracy throughout the trial. Fig S2 shows TV-RNNs with *N* = 16 (black curve), which have a comparable number of parameters to a standard RNN with *N* = 64. The results indicate that TV-RNNs with fewer hidden states also perform well in temporal classification. This demonstrates that the improved performance of TV-RNNs is not merely due to an increased number of parameters compared to standard RNNs. This paper focuses on models with an equal number of hidden states to ensure consistency in the hidden space across models.

Standard RNNs may suffer from the vanishing and exploding gradient problems since, during BPTT, gradients of earlier time steps are obtained by repeatedly multiplying through static weight matrices, as detailed below. To distinguish recurrent weights of standard RNNs and single recurrent weights of TV-RNNs, we use ${\overset{ˆ}{W}}_{h}$ to represent recurrent weights of standard RNNs.

(12) $$\frac{\partial L}{\partial {\overset{ˆ}{W}}_{h}}=\frac{1}{T}\sum_{t=1}^{T}\frac{\partial l_{t}}{\partial y_{t}}\frac{\partial y_{t}}{\partial h_{t}}\frac{\partial h_{t}}{\partial {\overset{ˆ}{W}}_{h}}$$

(13) $$\frac{\partial h_{t}}{\partial {\overset{ˆ}{W}}_{h}}=\frac{\partial f\left(x_{t},\;h_{t-1},\;{\overset{ˆ}{W}}_{h}\right)}{\partial {\overset{ˆ}{W}}_{h}}\;\;+\sum_{i=1}^{t-1}(\prod_{j=i+1}^{t}\frac{f\left(x_{j},\;h_{j-1},\;{\overset{ˆ}{W}}_{h}\right)}{\partial h_{j-1}})\frac{f\left(x_{i},\;h_{i-1},\;{\overset{ˆ}{W}}_{h}\right)}{\partial {\overset{ˆ}{W}}_{h}}$$

where

(14) $$f\left(x_{t},h_{t-1},{\overset{ˆ}{W}}_{h}\right)=tanh\left({\overset{ˆ}{W}}_{h}h_{t-1}+W_{x}x_{t}+b_{h}\right)$$

The term $\prod_{j=i+1}^{t}\frac{f\left(x_{j}h_{j-1},\;{\overset{ˆ}{W}}_{k}\right)}{\partial h_{j-1}}\propto \prod_{j=i+1}^{t}{\overset{ˆ}{W}}_{h}$ causes the gradient vanishing and exploding, especially when T is large. For instance, when the recurrent weights have small eigenvalues α<1, the gradients shrink exponentially, leading to the vanishing gradient problem, making long-term dependencies hard to learn. Conversely, if the weights have large eigenvalues α>1, the gradients grow exponentially, causing the exploding gradient problem, which destabilizes training. With an example length of sequence [i+1,t], the gradient multiplication of standard RNN is defined as follows. For simplicity of notation, we define $t^{\prime }=i+1$

(15) $$J_{RNN}\left(t^{\prime },\;t\right)=\;\prod_{j=l^{\prime }}^{t}W_{h}={\left({\overset{ˆ}{W}}_{h}\right)}^{t-l^{\prime }}$$

In contrast, TV-RNNs use multiple distinct recurrent weights, leading to the term $\prod_{j=l^{\prime }}^{t}\frac{f\left(x_{j},h_{j-1},W_{h}^{t}\right)}{\partial h_{j-1}}\propto \prod_{j=l^{\prime }}^{t}W_{h}^{t}$ being composed of varying weight matrices. The gradient multiplication of standard RNN is defined as:

(16) $$J_{TV}\left(t^{\prime },t\right)=\prod_{j=t^{\prime }}^{t}\left(W_{h}^{t}\right)$$

Unlike standard RNNs, where a single weight matrix leads to uniformly large or small eigenvalues, TV-RNNs allow different eigenvalues across time steps. According to Multiplicative Ergodic Theorem [44], there exists a Lyapunov exponent λ such that

(17) $$\underset{t-t^{\prime }\to \infty }{lim\;sup}\frac{1}{t-t^{\prime }}log\|J_{TV}\left(t^{\prime },t\right)\;=\lambda$$

where $t^{\prime }=i+1$. Here, we use the L1 norm, since the magnitude is important in preventing gradient vanishing or explosion. Thus,

(18) $$\left\|J_{TV}\left(t^{\prime },t\right)\right\|=\prod_{j=t^{\prime }}^{t}\left(W_{h}^{t}\right)\approx e^{\lambda \left(t-t^{\prime }\right)}$$

Since the operator norm is submultiplicative, we have

(19) $$\lambda \leq E\left[log\left\|W_{h}^{t}\right\|\right]$$

then, $W_{h}^{L}$ and $W_{h}$ can be considered sampled from the same distribution, therefore, we have

(20) $$\|J_{TV}\left(t^{\prime },t\right)\|\leq e^{\left.E\left[log\left\|W_{h}^{t}\right\|\right]\right\}\left(t-t^{\prime }\right)}=e^{log\left(\left\|W_{h}\right\|\right)\left(t-t^{\prime }\right)}$$

So

(21) $$\left\|J_{TV}\left(t^{\prime },t\right)\right\|\leq \left\|J_{RNN}\left(t^{\prime },t\right)\right\|$$

In summary, gradients grow or vanish exponentially in standard RNNs due to repeated multiplication of the same weight matrix, whereas in TV-RNNs, time-varying weights cause gradients to evolve at a slower rate due to the variability of the weight matrices. This variability prevents the consistent amplification or suppression of gradients, helping overcome issues regarding vanishing and exploding gradients. The visualization of the gradients of an example recurrent weight across first 1000 training epochs of RNN-S2 is shown in Fig 4B (see Fig S1A for another example) and the gradients of an example recurrent weight in first window of TV-RNNs are shown in Fig 4C. Moreover, the histogram of gradients is shown in Fig 4D. Later example windows are shown in Fig S1C and S1D. As an additional control, gradients of RNN-S2 initialized by RNN-S1 are shown in Fig S1B, which is a strategy used to train TV-RNNs. The results show that RNN-S2 suffers from the common gradient vanishing and exploding problem, resulting in prolonged training. In contrast, TV-RNNs reduce the likelihood of gradient vanishing and exploding, enabling more stable and efficient training. Here, the Transformer architecture is also applied, with results presented in the supplementary tables and figures. Overall, the Transformer performs comparably to TV-RNNs, with notably better performance at earlier time points. This advantage is more evident in simulated datasets with abundant trials. In contrast, for real neuroscience datasets where data is limited (see next), TV-RNNs outperform Transformers.

#### 2) WFCI Dataset:

**Fig. 5.**
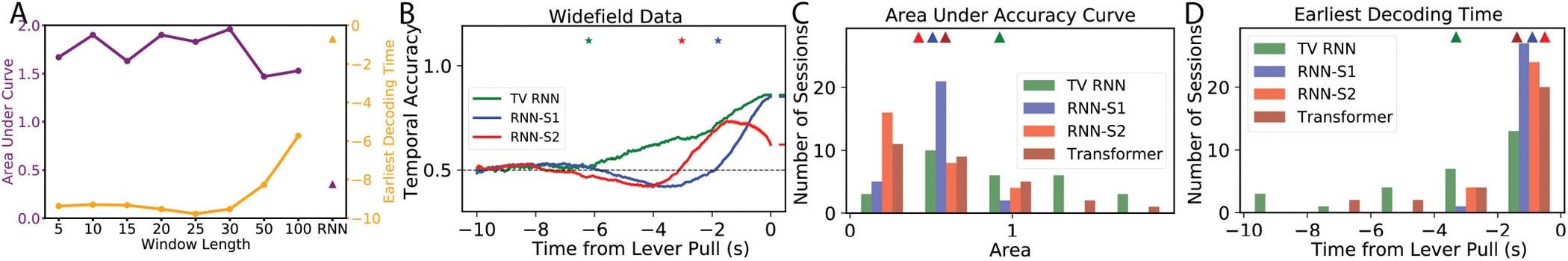
(A) Determining the window size w of TV-RNN: area under curve and earliest decoding time (see Methods) while varying w from 6 to 30; triangles represent standard RNN. (B) Temporal accuracy of standard RNNs with two training strategies and TV-RNNs, the stars depict the earliest decoding time with the height representing the sequential classification accuracy. (C) Histogram of the area under accuracy curve using standard RNNs and TV-RNNs for all sessions of mouse. (D) Histogram of the earliest decoding time using standard RNNs and TV-RNNs for all sessions of mouse.

Standard RNNs and TV-RNNs are trained to classify the WFCI dataset of 300 time points, i.e., from 10 seconds before the behavior (lever pull) to the time that the behavior happens (see Methods), in order to quantify the earliest behavioral decoding time and the temporal performance of decoding with real data. An example session of one mouse with a large number of trials (here, 378 trials) is used to optimize the window length w between the values of 5 and 100, while computing the AUAC and earliest decoding time as metrics of interest (Fig 5A). The setting w=30 achieved the highest AUAC and offered a favorable trade-off between model simplicity and early decoding. Thus, the TV-RNN w is set to 30, which implies that every 30 time points (1 second) will lead to a switch in the weights. Fig 5B shows the temporal classification accuracy with combined trials (2447 *‘behavior’* trials). The results show that the time around lever pull has the highest accuracy in both standard RNNs with S1 training strategy and TV-RNNs. Using standard RNNs, the behavior can be classified significantly above chance up to around several seconds prior to the lever pull, i.e., around 2 seconds in S1 and around 3 seconds in S2. This also illustrates that S2 performs better than S1 in early classification but worse in final classification, i.e., around 0.85 in S1 and 0.62 in S2. The results also show that TV-RNNs significantly outperform standard RNNs in most time points in Fig 5B. Importantly, the earliest decoding time of TV-RNNs can reach around 6 seconds before the lever pull, and the final classification accuracy is around 0.86. The temporal accuracy curves of standard RNNs and TV-RNNs for another 5 mice with combined trials are shown in Fig S3. The TV-RNNs outperform standard RNNs in most time points as well, and all TV-RNNs have the earliest decoding time reach around 6 to 8 seconds before the lever pull. Additionally, a modern architecture, i.e., Transformer, is applied on the same dataset (see Methods for causal Transformer). As shown in Table I, Transformers perform comparably to the proposed TV-RNNs when sufficient training trials are available. However, with limited data, their performance declines, and TV-RNNs outperform them (see below).

Next, the session-by-session accuracy of the TV-RNNs is evaluated. Fig 5C shows the AUAC for selected sessions (with #trials ≥ 40). The results consistently show that TV-RNNs achieve a higher accuracy than standard RNNs, presumably because TV-RNNs explicitly take into account more temporal structure with time-varying weights in the model, and allow for a monotonically increasing classification accuracy. Finally, the earliest decoding time is shown in Fig 5D, where TV-RNNs outperform standard RNNs in most sessions of the example mouse. Additionally, in this single-session comparison with fewer trials, TV-RNNs outperform the Transformer, demonstrating their ability to perform well under limited data.

The temporal classification performance of WFCI dataset between TV-RNNs and other classifiers are compared next. 10 standard RNNs are trained independently with each taking 30 time points (1 second) of the entire 300 time points (10 seconds before the lever pull). All the 10 standard RNNs have initialized hidden states of zeros. Note, these are different from TV-RNNs in which all sets of weights are dependent because of the continuous hidden states. Additionally, 10 standard RNNs are trained by using strategies RNN-S1 and RNN-S2 seperately as well. Fig S5 shows the temporal classification accuracy of TV-RNNs and independent standard RNNs with combined trials for all 6 mice. The results show that independent standard RNNs with S1 training strategy (purple curves) have large oscillations. This time, the earliest decoding time of all the mice is around 1 second before the lever pull, and the final accuracy of them is lower than the final accuracy of TV-RNNs. This matches the finding of standard RNNs with S1 training strategy in Fig 5B, i.e., standard RNNs trained by using the loss at the end of the sequence are not able to classify the sequence accurately at early time. In contrast, independent standard RNNs with S2 training strategy (orange curves) can classify accurately at early time but not as accurately as TV-RNNs at final time. The AUAC of TV-RNNs and independent standard RNNs is shown in Fig S7, it shows that TV-RNNs outperform independent standard RNNs at most time points, which illustrates that TV-RNNs do better not only because they have more weights than standard RNNs.

TV-RNNs are compared with SVMs, which are built using as input 1-s-wide windows (30 time points) of data. The input to each SVM classifier was a 2D array, i.e., [#*trials*,#*timepoints* * #*components*] [5]. The classification accuracy of each window is reported as the temporal classification accuracy of the last time point in this window. SVMs are trained with linear kernel and radial basis function kernel (RBF). This paper only shows the results of SVMs with RBF kernel in Fig S6 because of its better performance than a linear kernel. The SVMs outperform TV-RNNs in earliest decoding time but have lower final accuracy than TV-RNNs. The AUAC of TV-RNNs and SVMs are comparable. Indeed, sliding SVMs have 300 classifiers with each of them trained for 1 time point specifically, thus, it is fitted to each time point, especially at the early time points which have rare behaviorally-relevant information. Additionally, the classification accuracy after 2 seconds from the earliest decoding time is only slightly above the chance level. Furthermore, TV-RNNs outperforming sliding SVMs at final accuracy reveals that TV-RNNs can accumulate information for classification which multiple independent classifiers cannot.

#### 3) fMRI Dataset:

**Fig. 6.**
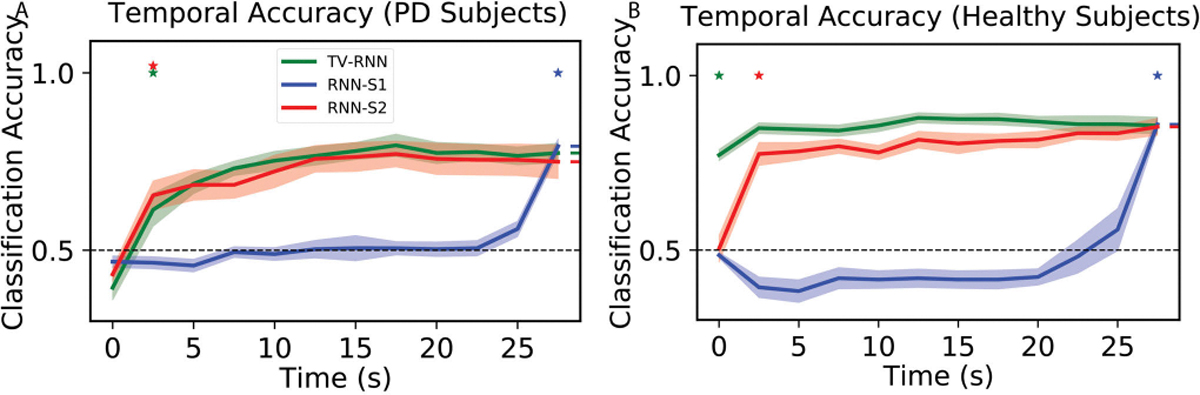
(A) Temporal accuracy of behavioral classification between ‘force’ and ‘rest’ for PD patients; (B) Temporal accuracy of behavioral classification between ‘force’ and ‘rest’ for healthy control.

In order to test the performance of TV-RNNs on the fMRI dataset, the same methods are applied towards data from PD patients and healthy controls (see Methods). After exploring the best w of the TV-RNNs using the same methodology as for the WFCI dataset, w is set to 2 timepoints for this fMRI dataset. The standard RNNs and TV-RNNs are trained by using the neural signal recorded from PD patients; the temporal accuracy curves are shown in Fig 6A. Here, TV-RNNs are able to not only classify the sequence accurately at the end but also achieve early classification, at around 2.5 seconds after the beginning of the sequence. Moreover, the temporal accuracy curves of healthy subjects in Fig 6B illustrate that TV-RNNs outperform standard RNNs and can achieve accurate classification at extremely early times, i.e., at the beginning of the sequence. Similarly, classification results using the Transformer are presented in Fig. S4 of the Supplementary Materials, where the Transformers underperformed on the fMRI dataset with a limited number of trials. For the fMRI data, unlike for the simulated and WFCI data, the standard RNN-S2 is able to predict correctly at a relatively early time and keep high final accuracy in both PD patients and healthy control. One possible reason is that the subject starts applying a grip force from the beginning of the task, and the length of the sequence is shorter (only 12 time points), and thus standard RNNs are able to memorize most of the previous input. Another potential reason is that, as is evident in the STFT magnitude (Fig 3F), the data statistics are stable across time unlike the distributional shifts present in widefield datasets. Lastly, the classification accuracy is higher in healthy subjects than in PD patients, which can give us the insight that healthy subjects perform the finger moving task better than PD patients, who usually have a tremor in one hand.

TV-RNNs can be regarded as the best choice in early and accurate classification because of their best overall performance, as they can achieve both early and accurate sequential classification. The ability to classify behavior early from neural activity holds significant clinical promise. In Parkinson’s disease, such early detection could support closed-loop neuromodulation systems that deliver targeted stimulation before its onset. Moreover, identifying distinct temporal patterns in neural dynamics may contribute to the development of robust biomarkers for early diagnosis and monitoring of neurodegenerative diseases.

### C. Classification Mechanisms

**Fig. 7.**
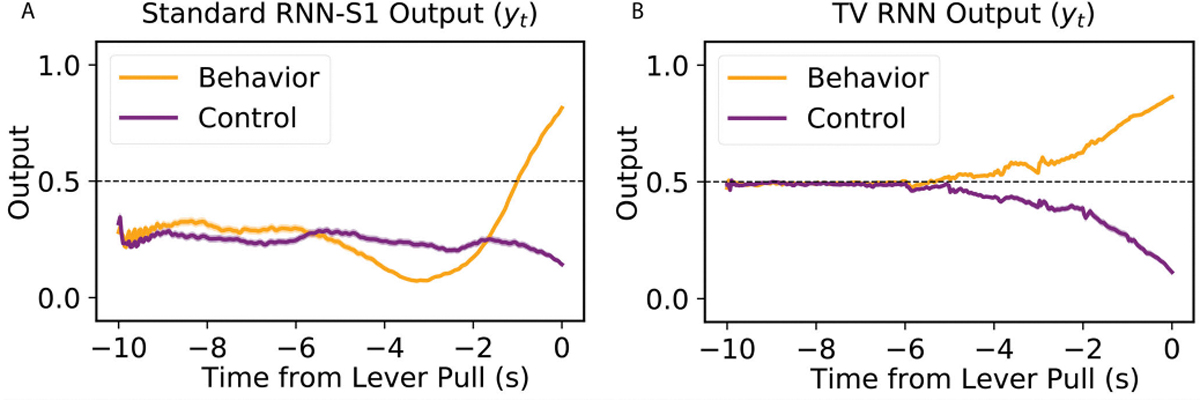
(A) Output trajectories of standard RNNs (average across trials), in the WFCI data. The shaded region provides the standard deviation. (B) Similarly, the output trajectories of TV-RNNs.

In order to understand how the exact output of RNNs changes with shifting data distributions, the trained network (for widefield dataset) activity is succinctly visualized: the output of the networks y(t) is shown in Fig 7A and B. The RNN output trajectories (Fig 7A) starts to diverge between the two classes at an early time, and at around 2 seconds before the behavior, the trajectories from the two classes start to diverge quickly. Thus, the evidence for decision making between the two classes does not exist in the output nodes until close to the final time step *T*, at which point the information moves from the memory to the output nodes and the classification is performed. On the contrary, in Fig 7B, the TV-RNNs output trajectories start diverging at the beginning and towards the decision with accumulation of evidence [45], the *‘behavior’* and *‘control’* trajectories start to diverge at around 5 seconds before the behavior.

The same output trajectories for the simulated data and fMRI data are shown in supplementary Fig S8, Fig S9, and Fig S10. The standard RNN-S1 has overlapping trajectories between *‘behavior’* and *‘control’* at early times, and they cannot diverge well even near the end of the trial. The TV-RNNs output trajectories for simulated data and fMRI data have a similar tendency as the WFCI dataset: they also diverge at the beginning and towards the decision with accumulation of evidence. Therefore, the TV-RNNs seem to utilize the temporal features in the data to accumulate evidence in order to make a decision. This is the reason why TV-RNNs not only outperform standard RNNs in Fig 4, Fig 5B and Fig 6, but also why TV-RNNs are able to achieve monotonically increasing decoding.

### D. Prediction of Upcoming Behavior

The output trajectories $\left(y_{t}\right)$ of TV-RNNs applied to *‘behavior’* trials (orange curve in Fig 7B) show that TV-RNNs are capable of predicting the behavior happening in the future. In WFCI datasets, after approximately 6 seconds prior to the lever pull, the TV-RNNs can predict the *‘behavior’* trials above the threshold of 0.5 which means the TV-RNNs effectively identify the upcoming lever pull. Specifically, if given only 4 seconds of data, from −10s to −6s, the models can predict that a lever pull will happen 6 seconds in the future, which is better than chance level accuracy. This is also possible to perform at any time point, i.e., at $\tilde{t}$, the model is able to predict a lever pull happening $\left|\tilde{t}\right|$ seconds in the future. As a comparison, standard RNNs can only predict the upcoming behavior around 1 second before the behavior. TV-RNNs also outperform standard RNNs in prediction of upcoming behavior in other datasets (Fig S8B, Fig S9B, and Fig S10B).

### E. Analysis of Weights

**Fig. 8.**
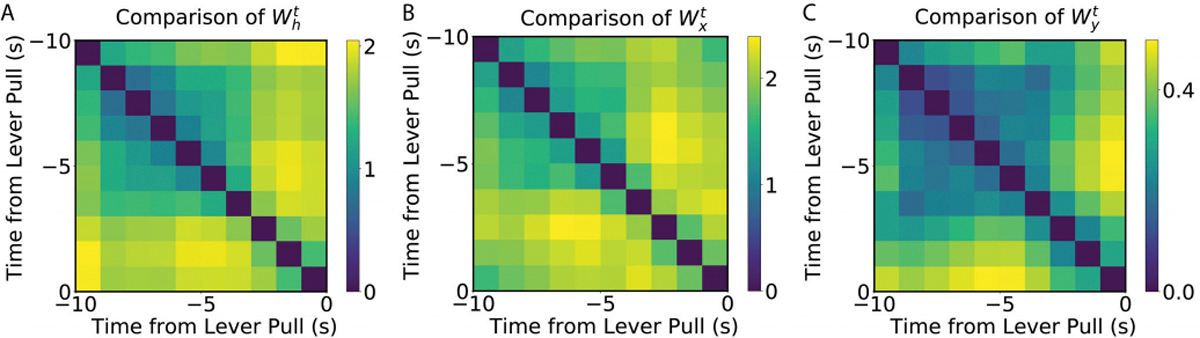
(A) Euclidean distance between $W_{h}^{t}$ of TV-RNN at different time. (B) Euclidean distance between $W_{x}^{t}$ of TV-RNN at different time. (C) Euclidean distance between $W_{y}^{t}$ of TV-RNN at different time.

In order to understand why TV-RNNs are more efficient at classification, and to analyze the difference between TV-RNNs and standard RNNs, the learnt time-varying weights across the trial are compared by calculating the euclidean distance between the time-varying weights, i.e., $W_{x}^{t},\;W_{h}^{t}$, and $W_{y}^{t}$, at different times, results are shown in Fig 8A, B, and C. Note that the color-map illustrates the euclidean distance, with brighter colors representing a larger difference. The weights are very different closer to the behavior. Furthermore, the recurrent weights $W_{h}^{t}$ in Fig 8A shows more changes than the other weights, which may be necessary here to exploit the dynamic nature of the temporal features. Moreover, the sharpest changes in the input and recurrent weights are at around 3 seconds before the behavior, which matches the changes in the signal statistics in the STFT (Fig 3E). The output weights $W_{y}^{t}$ do not have a large variation, which reveals that the divergence between the trajectories of the two classes already exists in RNN layers. Consequently, the output weights can distinguish two classes without many changes. The learnt time-varying weights across the trial for the simulated data and fMRI data are also compared (see Supplementary Figure 11–13). Fig S11 shows the change of the weights for simulated data; the sharpest changes in all the input, recurrent and output weights are at the beginning of the trial, and the changes decrease across the trial. This reflects that the TV-RNNs capture the information for classification at the beginning, and keep it until the end, and matches the findings from the temporal accuracy curve in Fig 4 green curve. Likewise, the fMRI data for both PD patients and healthy control shows sharpest change at the beginning, which also matches the temporal accuracy curve of fMRI data in Fig 6 (see Fig S12 for PD patients and Fig S13 for Healty subjects).

### F. Transfer Learning on WFCI Data

In order to test the generalization of the trained models across subjects, the transfer learning performance of the TV-RNNs is compared with that of standard RNNs. The model is trained on data from one mouse and tested on data recorded from another mouse. Fig S14 shows the across-subject temporal accuracy curves. As a comparison, the performance of within-subject models (dashed curves) is also shown. TV-RNNs outperform RNN-S1 in transfer learning across mice in most cases. The results show the difference between TV-RNNs and RNN-S1 in transfer learning quantitatively in Fig S15. TV-RNNs have better earliest decoding time in 96% of transfer cases, TV-RNNs also outperform RNN-S1 in AUAC in 90% of transfer cases. TV-RNNs and RNN-S1 have comparable final accuracy in transfer learning.

### G. Quantifying the Contribution of Different Regions

#### 1) Simulated Data:

The importance matrix of simulated data is shown by calculating the average absolute SHAP value of each feature across all trials in the test set of the datasets (see Methods for details). The matrix in Fig S16A recovers the structure built into the trials, i.e., the consistent presence of the peaks in different dimensions at sequential time windows determines which class is the output. In Fig S16B, the importance is almost the same across regions. Only a few time points at the end of the sequence show relatively high importance. However, the importance value is around 10^−6^. This is because that TV-RNN uses all the features at each region and time point, and all the features play a similar role in decoding. This points to a robust encoding of the behavior in the feature set. We also compare SHAP value with a simpler approach, i.e., occlusion map, in Fig S17, and we find that this approach is not able to detect significant features in this task.

#### 2) WFCI Dataset:

Next, the contribution of brain regions on ‘lever pull’ behavior with combined trials (the same mouse as Fig 5B) is examined. the importance from SHAP value is computed by using all the temporal outputs of TV-RNNs. Here five of them, i.e., 8 seconds, 6 seconds, 4 seconds, 2 seconds, and 0 second before the behavior respectively, are shown in Fig S18, which means the contribution of brain regions on classification at 8 seconds, 6 seconds, 4 seconds, 2 seconds, and 0 second before the behavior respectively. These five importance matrix reflect the characteristic of RNNs, the input is forwarded into RNNs temporally, and the features of the input fed into the models after the target output time do not influence the target output. In the brain region domain, the left somatosensory upper limb region and the right somatosensory lower limb region show more importance than other regions across time, these two regions are considered to receive feedback from the right paw of the mice as it pulls the lever and to keep their body in balance. The regions with high importance are not always the same among different mice, showing subject-to-subject variability for mice performing the same task; the right somatosensory barrel field region, the motor regions, and the left somatosensory lower limb regions also show importance for other mice.

**Fig. 9.**
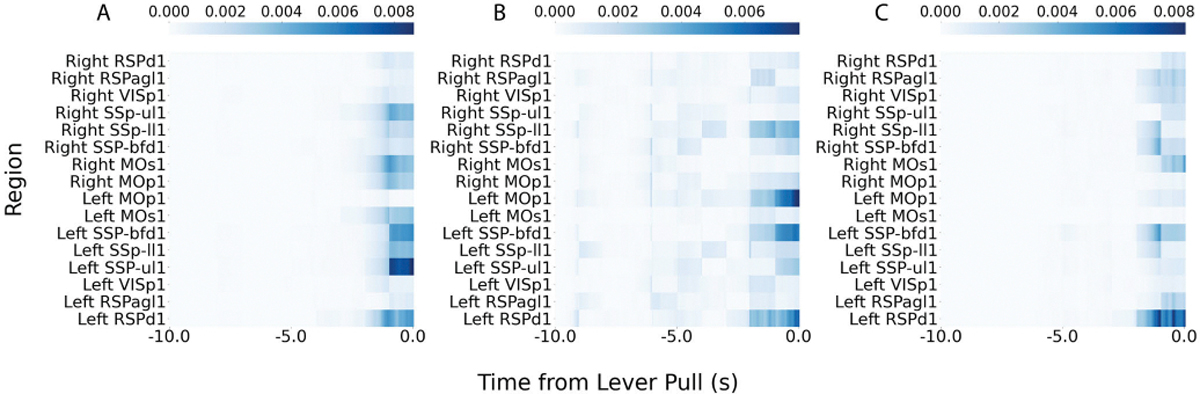
(A)(B)(C) Importance matrix of three example sessions with TV-RNN. The color represents importance based on SHAP values, with darker color indicating higher importance and lighter color indicating lower importance.

In Fig 9, the temporal importance of different brain regions is shown, while the behavioral classification of the example mouse performing the same self-initiated behavior is performed by TV-RNNs (Fig 9A, B, and C) and standard RNNs (not shown). According to the SHAP value at the end of the sequence, which is considered the final classification output, the somatosensory regions have more importance than other regions. Additionally, the motor area in dorsal part is also important in one example session (Fig 9B). The left regions show more importance than the right regions (Fig 9A and C), as all the mice used their right paw to pull the lever. The mice used their left paw to keep their body in balance when they pull the lever, so importance appears in the right regions as well. Moreover, the results show that when the mice used their paw to pull the lever, their lower limbs also moved, which may explain the finding that the somatosensory lower limb region is shown as important. The importance matrix of standard RNNs indicate similar important regions as the TV-RNNs case but less variable across time. This matches the mechanism of standard RNN and TV-RNNs.

#### 3) fMRI Data:

**Fig. 10.**
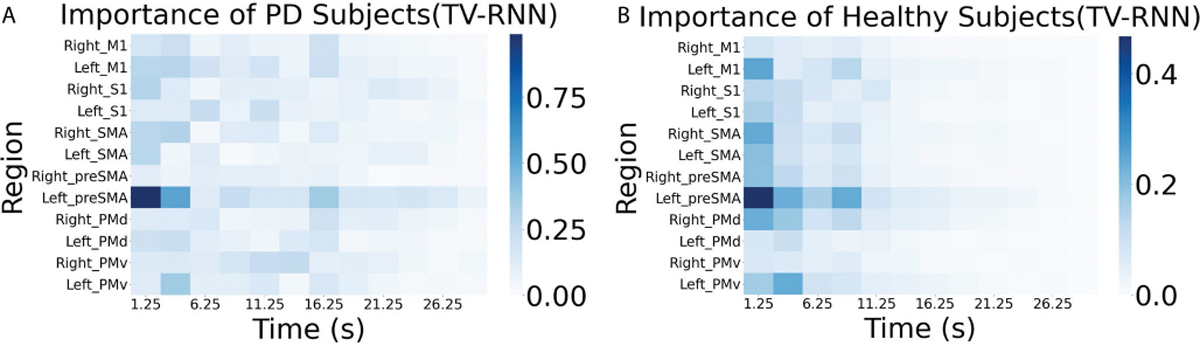
(A) Importance matrix of PD subjects with TV-RNNs; (B) Importance matrix of healthy subjects with TV-RNN.

The importance matrix of the fMRI dataset with TV-RNNs is shown in Fig 10A,B. Here, the left pre-supplementary motor area (Left preSMA) is shown to be most important for both PD and healthy subjects. However, in healthy subjects, the difference of importance between Left preSMA and the other regions is not as much as the case of PD subjects. Additionally, the most important regions for decoding are at the beginning of the sequence, which is different from the WFCI dataset.

### H. Computational Resources

On an example session, TV-RNNs require approximately 23.9 seconds per training epoch, compared to 10.8 seconds for RNN-S1, 10.9 seconds for RNN-S2, and 6.3 seconds for the Transformer model. In terms of memory usage, TV-RNNs, RNN-S1, and RNN-S2 each require about 5.22 GB to load the model in A100 GPU, while the Transformer demands 9.38 GB due to its substantially larger number of parameters. Although TV-RNNs require higher training time, they maintain memory efficiency and provide improved performance, making them a favorable trade-off for applications where accuracy is prioritized over training speed.

## V. CONCLUSION AND DISCUSSION

In this work, a novel time-varying model based on RNNs is developed to explore robust early sequential classification with brain-wide neural activity when the data distribution shifts across time. The results show that TV-RNNs are able to achieve temporal robust classification earlier than standard RNNs and have higher accuracy. This work also demonstrates that TV-RNNs have better transfer learning performance across different subjects. However, TV-RNNs may require longer time and more resources to train compared with standard RNNs and sliding SVMs. Moreover, TV-RNNs present challenges in tuning hyperparameters such as temporal window size w. This gives us a potential future direction on finding the optimal number of windows to use in TV-RNNs. Additionally, these models are able to reduce the probability of gradient vanishing and exploding, which standard RNNs suffer from. Moreover, SHAP values are used to quantify the importance of different regions. Results show somatosensory and motor areas at several seconds before the behavior are more important in behavioral decoding. The PreSMA region shows importance in both Parkinson’s patients and healthy controls. While the current study focuses on cross-subject generalization within the same modality, exploring cross-species and cross-modality transfer (e.g., fMRI to WFCI) is an important direction for future work. Such extensions would further test the model’s task-agnostic utility and its adaptability to diverse neural data. Future works also aim at robust online decoding using time series neural data for rehabilitation applications.

## Supplementary Material

supp1-3586175

This article has supplementary downloadable material available at https://doi.org/10.1109/TNSRE.2025.3586175, provided by the authors.

## Figures and Tables

**TABLE I:** Performance Comparison Across Different Models on WFCI

Model	Final Accuracy	EDT	AUAC

**TV-RNN (Ours)**	**0.82 ± 0.03**	**−6.6 ± 0.79**	0.959 ± 0.09
RNN-S1	0.81 ± 0.03	−1.59 ± 0.35	0.32 ± 0.07
RNN-S2	0.60 ± 0.03	−2.48 ± 1.16	0.48 ± 0.04
Transformer	0.80 ± 0.03	−6.49 ± 1.88	**0.962 ± 0.14**
