# Supplementary material for: Behavioral Classification of Sequential Neural Activity Using Time Varying Recurrent Neural Networks
Source: IEEE Trans Neural Syst Rehabil Eng. Author manuscript; Available in PMC 2025 Aug 30. (PMC12398402; doi:10.1109/TNSRE.2025.3586175)
Supplement: supp1-3586175 [file NIHMS2097780-supplement-supp1-3586175.pdf]

Supplementary Materials in ‘Behavioral Classification of Sequential Neural Activity Using Time Varying Recurrent Neural Networks’

Yongxu Zhang, Catalin Mitelut, David J. Arpin, David Vaillancourt, Timothy Murphy, Shreya Saxena

Algorithm 1 RNN-S1

standard RNN weights W^{S1}=$\{W_x,\ W_h,\ W_y,\ b_h,\ b_y\}$
for $iteration = 1, 2, \dots$ **do**
  for $batch = 1, 2, \dots, Max$ **do**
    input the time-series data to standard RNNs (see Equation 1 and 2)
    compute binary cross entropy loss at the end of sequence $L = l_T$
    BPTT with L
    Adam optimization $W^{S1}_{old} \leftarrow W^{S1}$
  end for
end for

Algorithm 2 RNN-S2

standard RNN weights W^{S2}=$\{W_x,\ W_h,\ W_y,\ b_h,\ b_y\}$
for $iteration = 1, 2, \dots$ **do**
  for $batch = 1, 2, \dots, Max$ **do**
    input the time-series data to standard RNNs (see Equation 1 and 2)
    compute binary cross entropy loss at the end of sequence $L = \sum_{t=1}^{T} l_t$
    BPTT with L
    Adam optimization $W^{S2}_{old} \leftarrow W^{S2}$
  end for
end for

Algorithm 3 Time-varying RNN

window size w, time T
TV-RNN weights W^t=$\{W^t_x,\ W^t_h,\ W^t_y,\ b^t_h,\ b^t_y\} \in W^{1,2,\dots,\frac{T}{w}}$
initialize W^t=W^{S1} $\forall t$
for $iteration = 1, 2, \dots$ **do**
  for $batch = 1, 2, \dots, Max$ **do**
    input the time-series data to TV-RNNs (see Equation 4 and Equation 5)
    $W^t = W^k, \quad \forall t \in [(k-1)w, kw], k \in [1, \frac{T}{w}]$
    compute binary cross entropy loss L by using the sum of loss at each time step $L = \sum_{t=1}^{T} l_t$
    BPTT with L
    Adam optimization $W_{old} \leftarrow W$
  end for
end for

Model	Final Accuracy	EDT	AUAC
**TV-RNN (Ours)**	**1**	0.6%	4.54
RNN-S1	0.98	98.7%	0.28
RNN-S2	0.78	33.7%	2.52
Transformer (causal)	0.99	**0%**	**4.87**

TABLE I
PERFORMANCE COMPARISON ACROSS DIFFERENT MODELS ON SIMULATED DATA.

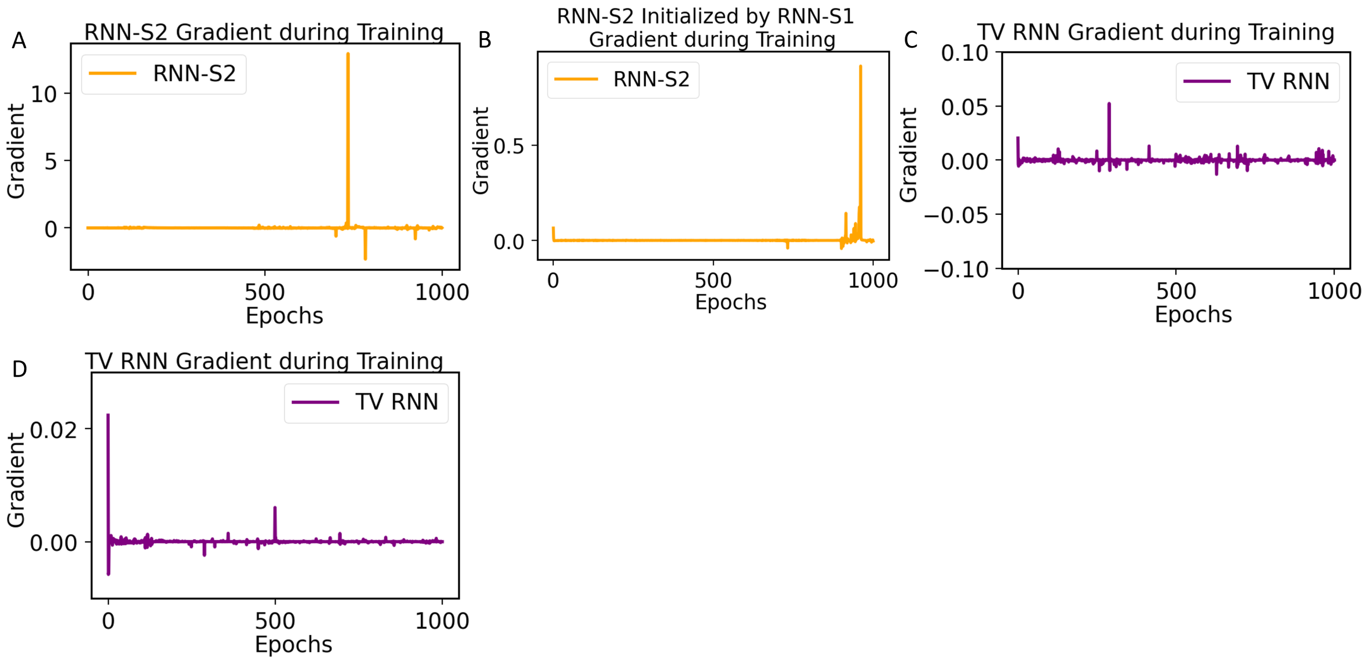

Fig. S1. Gradients of an example recurrent weight during training (first 1000 epochs) in (A) another run, (B) RNN-S2 initialized by RNN-S1, (C) second window of TV-RNN and (D) third window of TV-RNN. Please note the y-axis in (C) and (D) are different from (A) and (B).

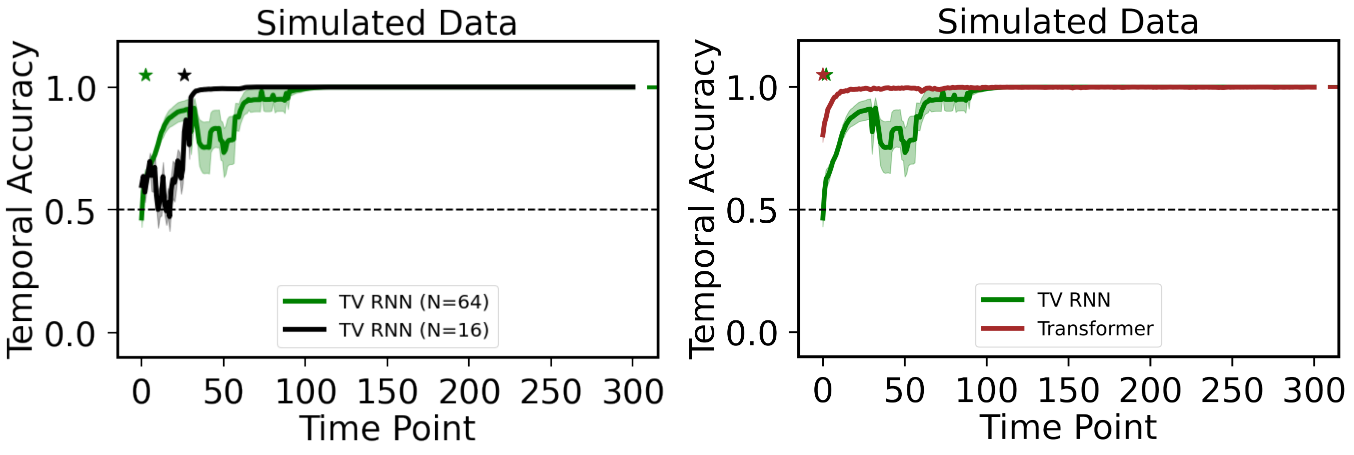

Fig. S2. Comparison between TV-RNNs with N=16 and N=64 on simulated data.

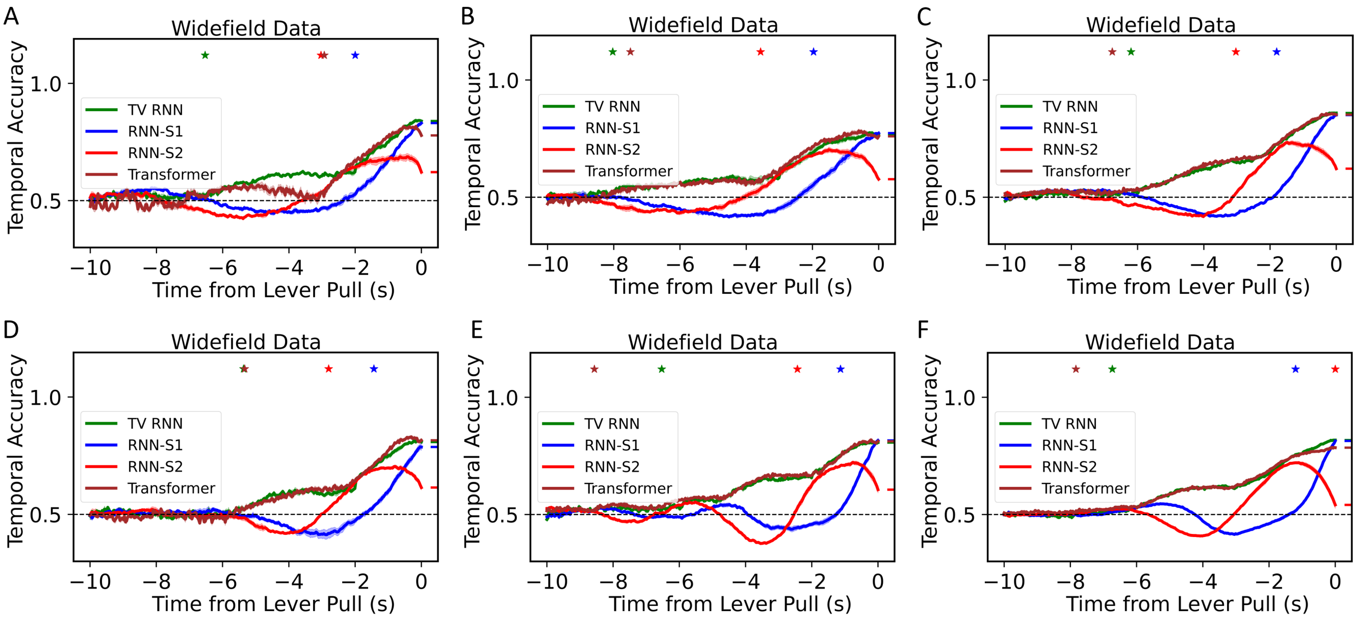

Fig. S3. Temporal accuracy of standard RNNs with two training strategies, Transformer (regular) and TV-RNNs for other 5 mice.

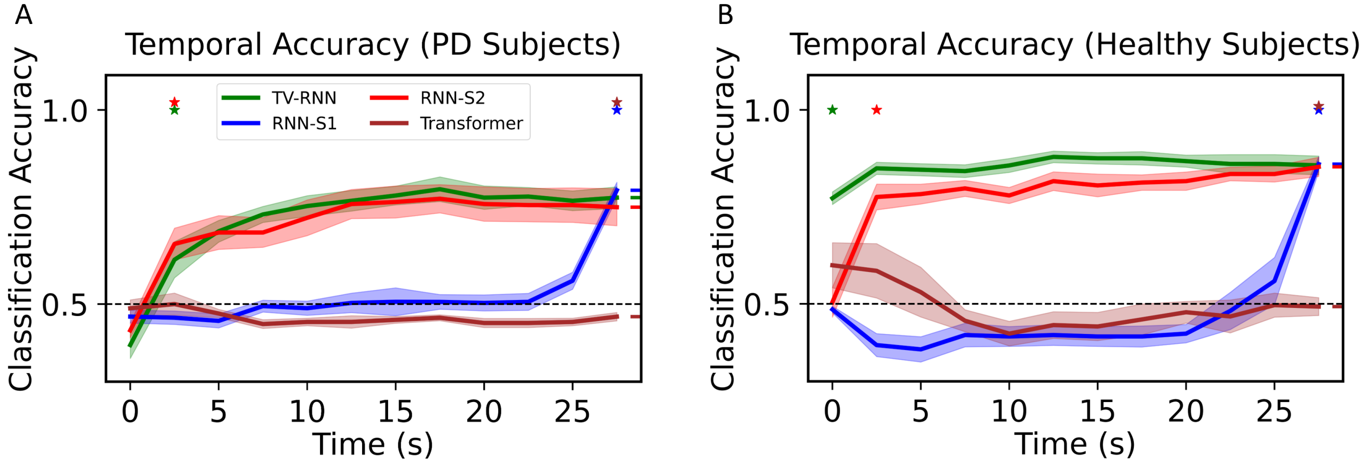

Fig. S4. Temporal accuracy of standard RNNs with two training strategies, Transformer (regular) and TV-RNNs for fMRI data.

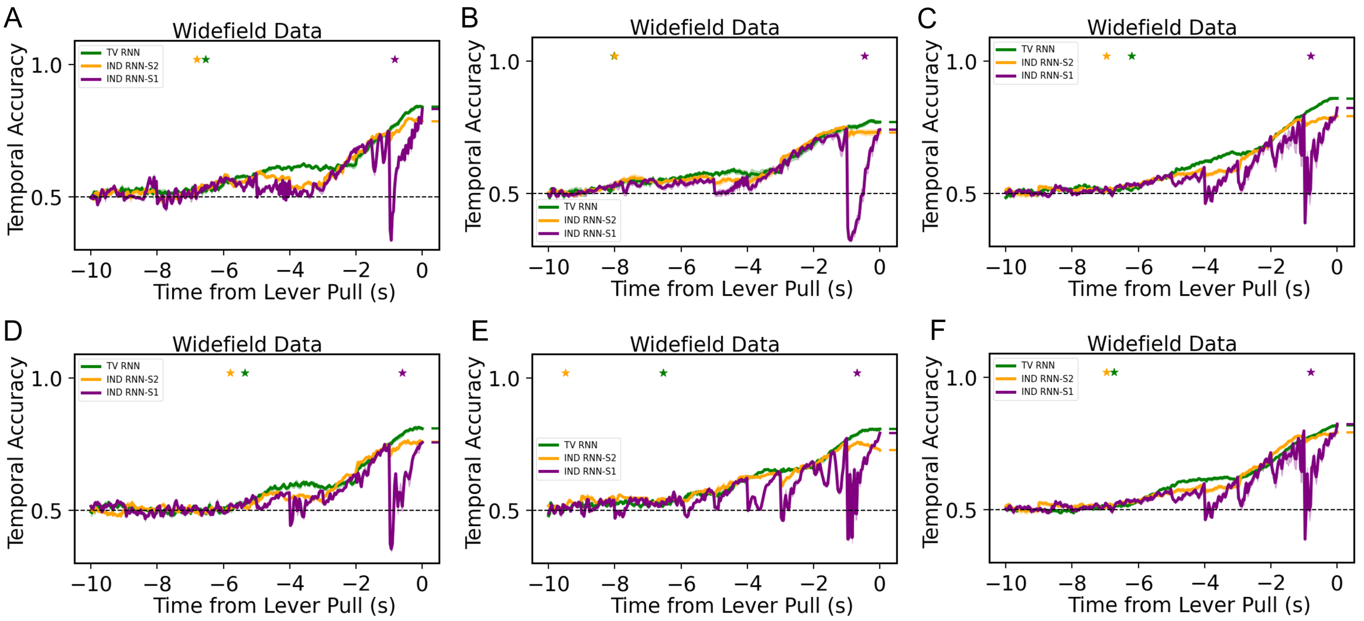

Fig. S5. Temporal accuracy of independent standard RNNs with two training strategies and TV-RNNs for all 6 mice.

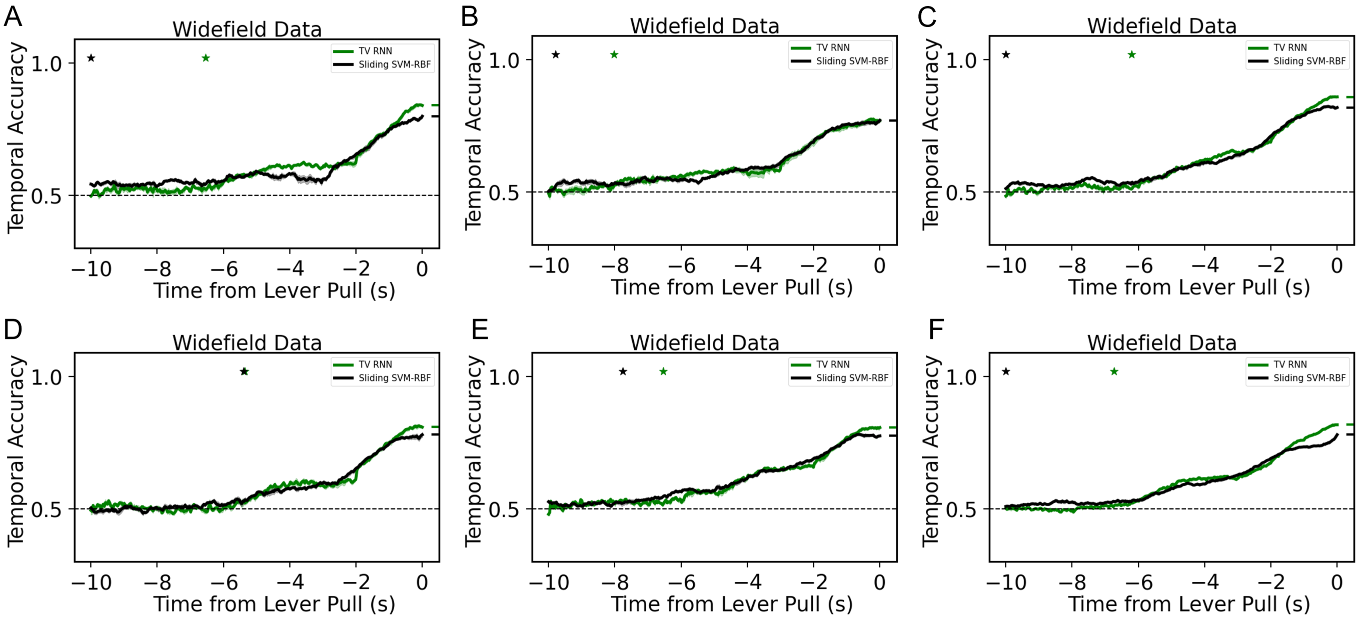

Fig. S6. Temporal accuracy of sliding SVMs and TV-RNNs for all 6 mice.

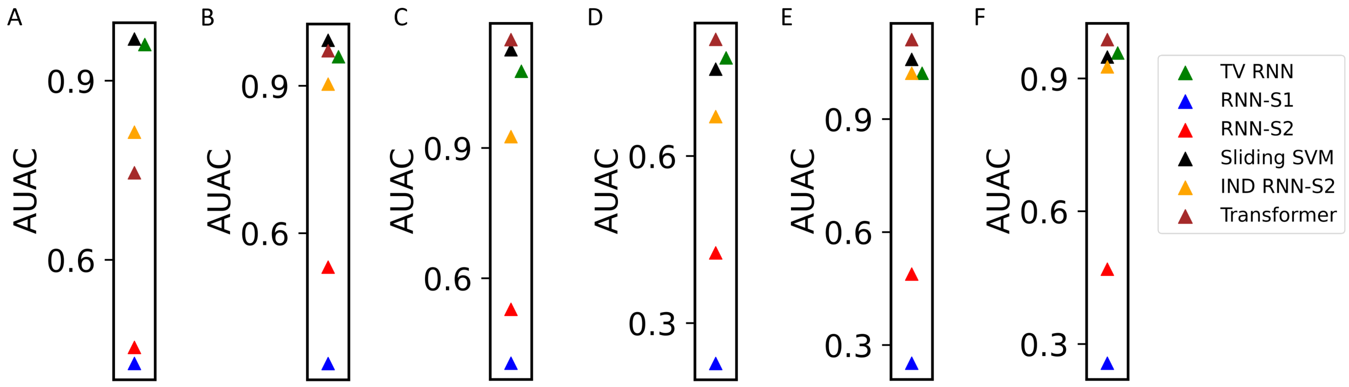

Fig. S7. Area under accuracy curve using all classifiers described above for all 6 mice.

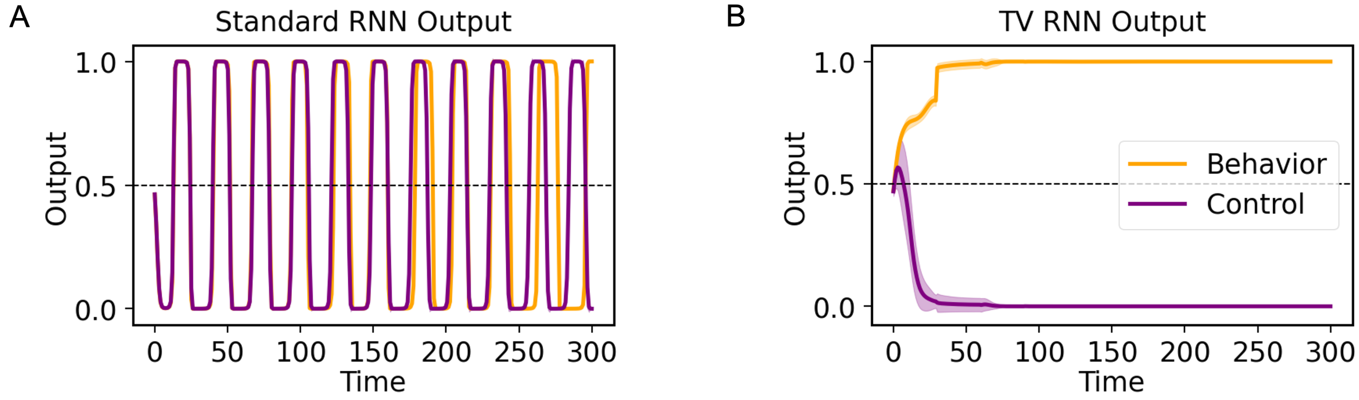

Fig. S8. (A) Output trajectories of standard RNNs (average across trials), in the simulated data. The shaded region provides the standard deviation. (B) Similarly, the output trajectories of TV-RNNs.

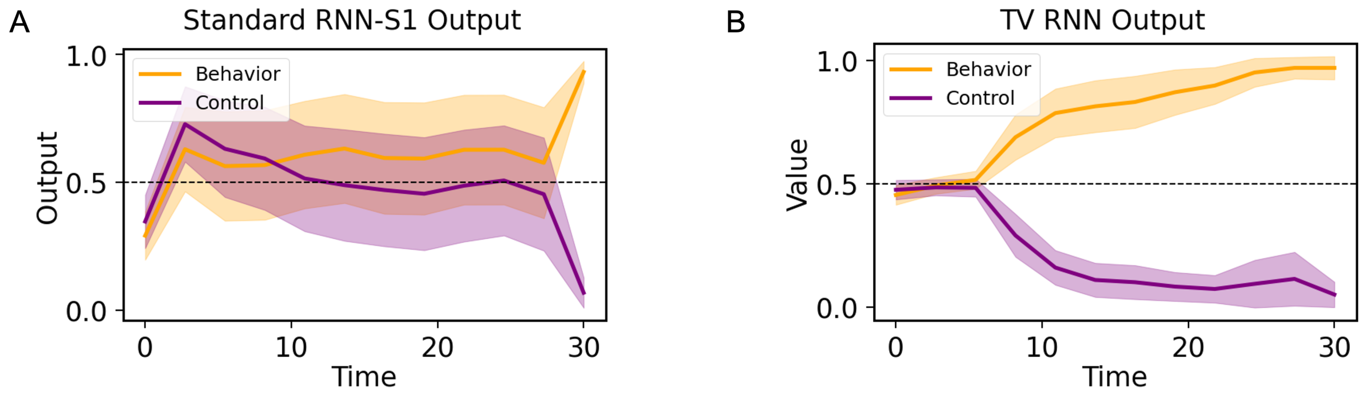

Fig. S9. (A) Output trajectories of standard RNNs (average across trials), in the fMRI data for PD patients. The shaded region provides the standard deviation. (B) Similarly, the output trajectories of TV-RNNs.

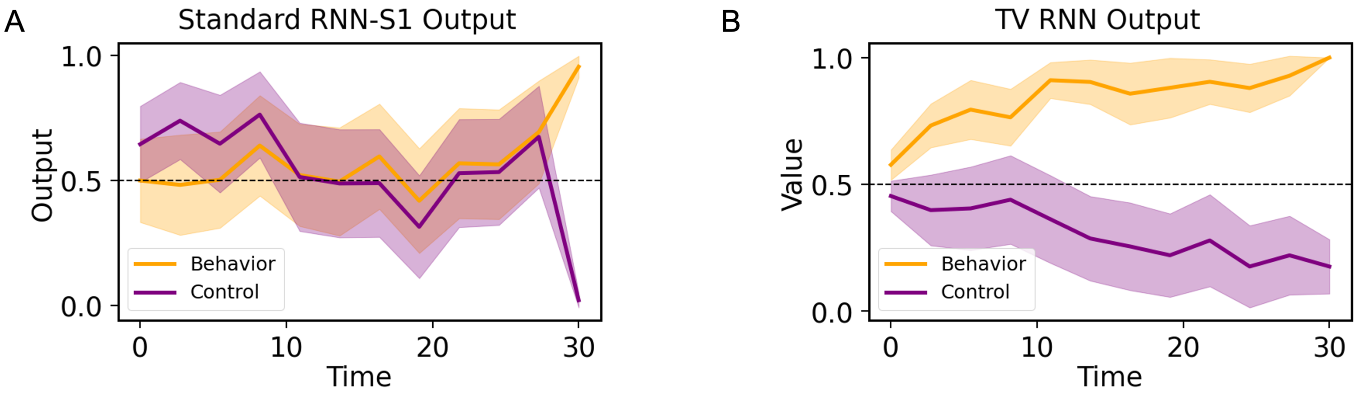

Fig. S10. (A) Output trajectories of standard RNNs (average across trials), in the fMRI data for healthy control. The shaded region provides the standard deviation. (B) Similarly, the output trajectories of TV-RNNs.

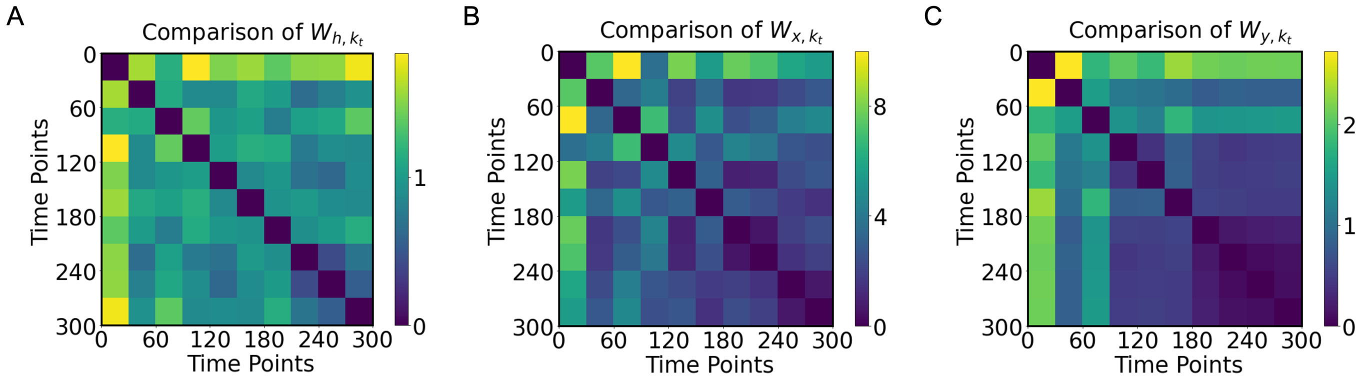

Fig. S11. Weights change of TV-RNNs in simulated data. (A) Euclidean distance between $\boldsymbol{W}_{\boldsymbol{h}}^{\boldsymbol{t}}$ of TV-RNN at different time. (B) Euclidean distance between $\boldsymbol{W}_{\boldsymbol{x}}^{\boldsymbol{t}}$ of TV-RNN at different time. (C) Euclidean distance between $\boldsymbol{W}_{\boldsymbol{y}}^{\boldsymbol{t}}$ of TV-RNN at different time.

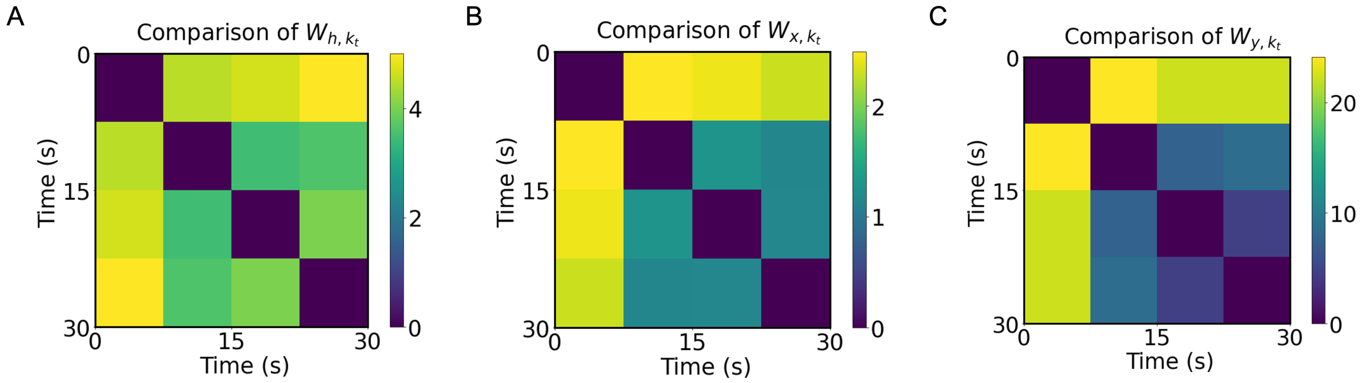

Fig. S12. Weights change of TV-RNNs in fMRI PD patient data. (A) Euclidean distance between $\boldsymbol{W}_{\boldsymbol{h}}^{\boldsymbol{t}}$ of TV-RNN at different time. (B) Euclidean distance between $\boldsymbol{W}_{\boldsymbol{x}}^{\boldsymbol{t}}$ of TV-RNN at different time. (C) Euclidean distance between $\boldsymbol{W}_{\boldsymbol{y}}^{\boldsymbol{t}}$ of TV-RNN at different time.

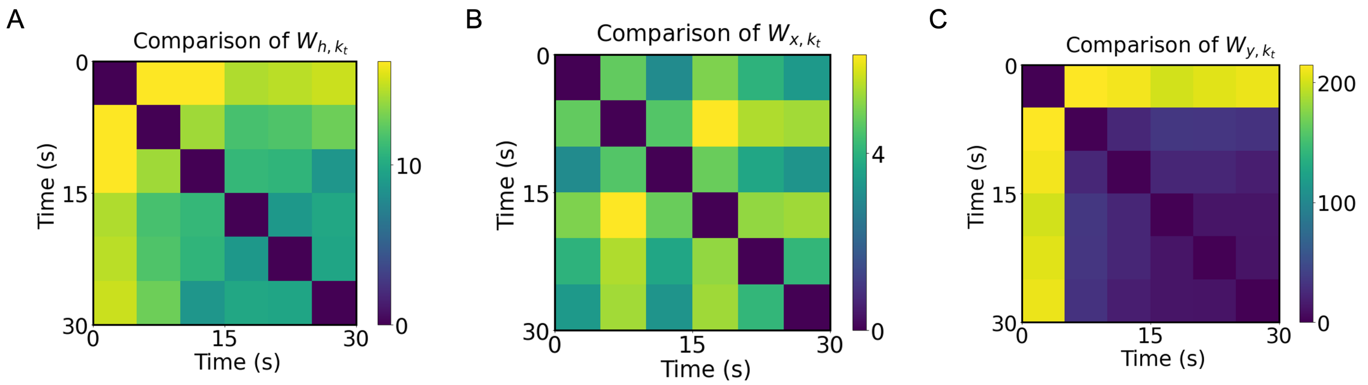

Fig. S13. Weights change of TV-RNNs in fMRI healthy control data. (A) Euclidean distance between $\boldsymbol{W}_h^t$ of TV-RNN at different time. (B) Euclidean distance between $\boldsymbol{W}_x^t$ of TV-RNN at different time. (C) Euclidean distance between $\boldsymbol{W}_y^t$ of TV-RNN at different time.

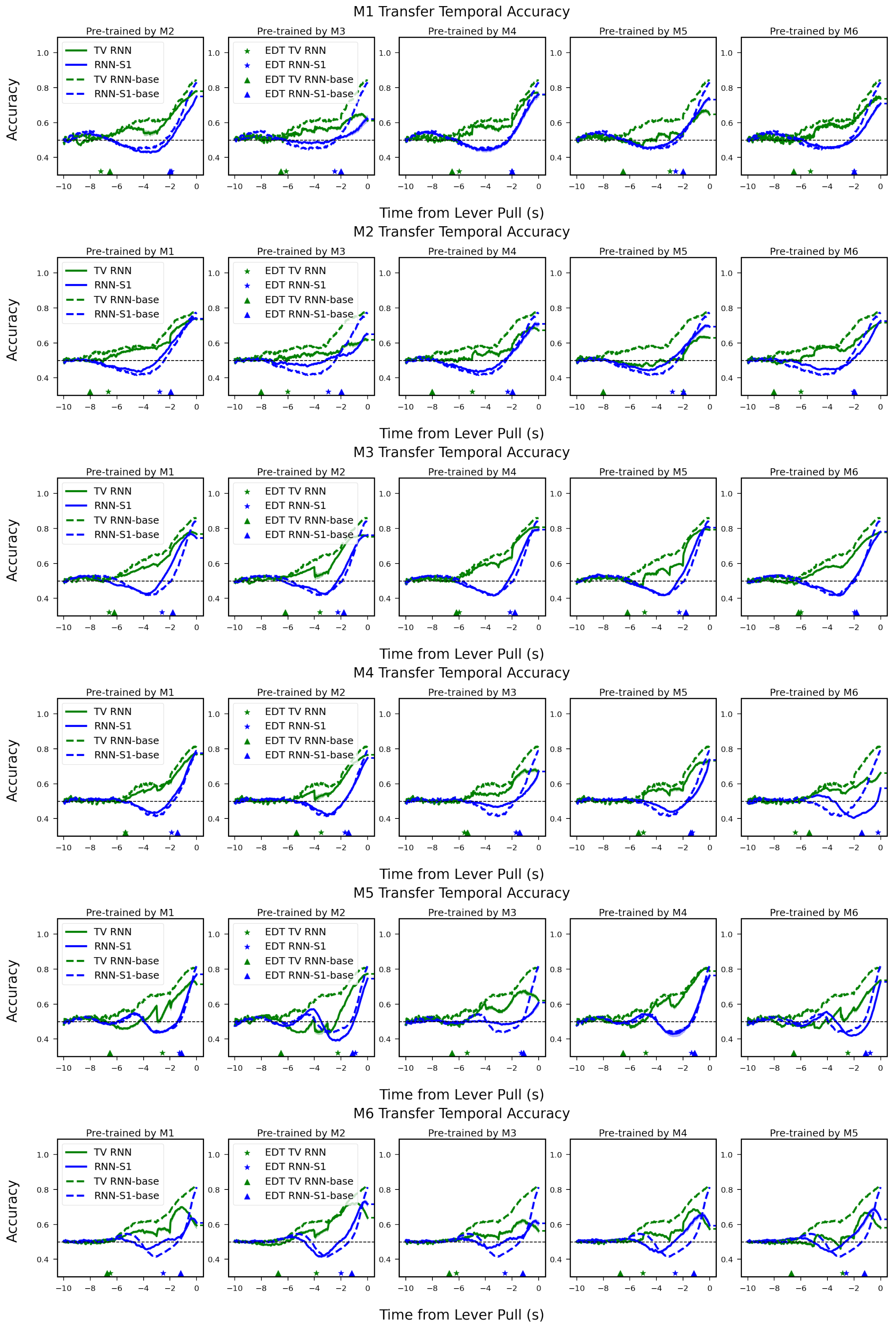

Fig. S14. Transfer learning performance of 6 mice. The dashed curves show the within-mouse classification performance (base), while the solid curves show the across-subject transfer learning performance.

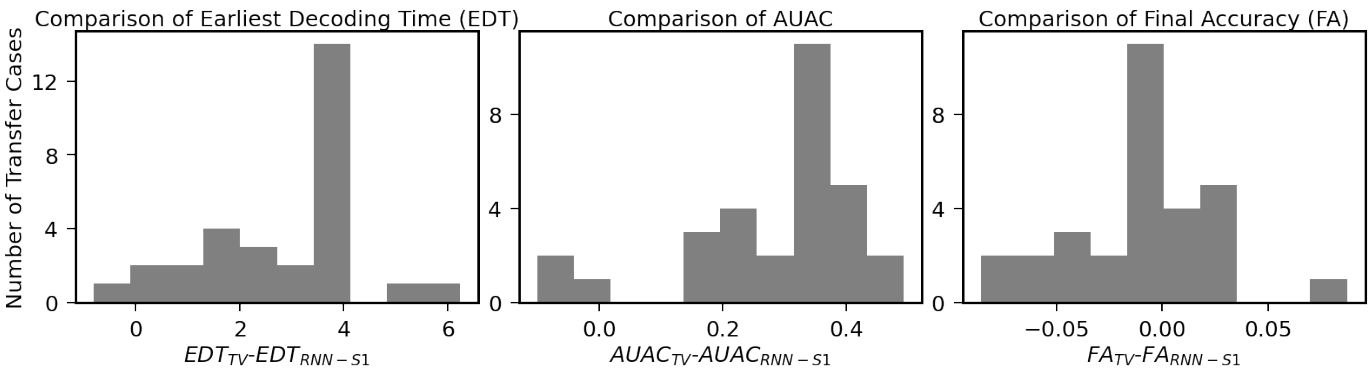

Fig. S15. Comparison of Earliest decoding time, AUAC, and Final accuracy between TV-RNNs and Standard RNNs in transfer learning.

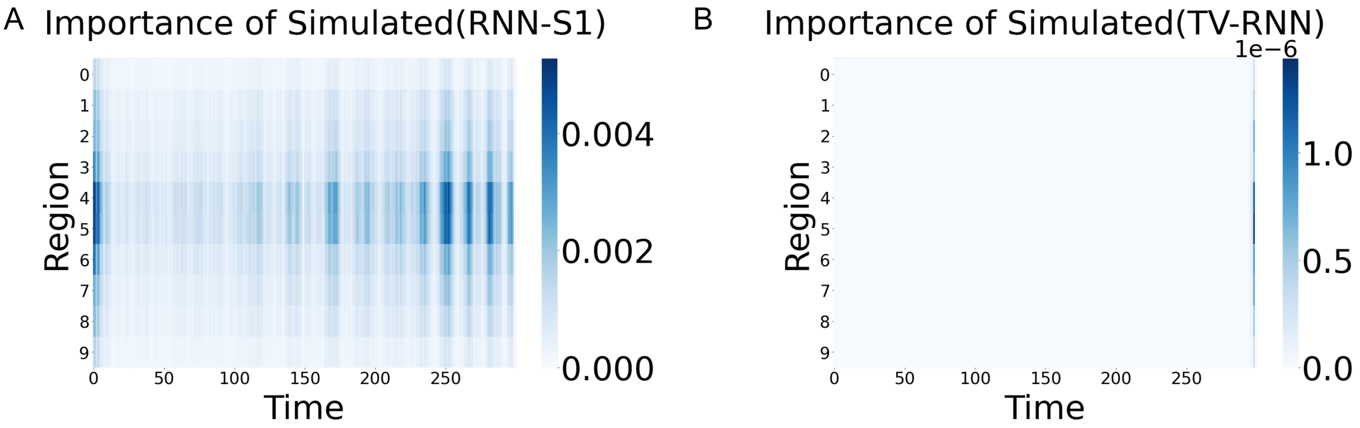

Fig. S16. (A) Importance matrix of simulated data with standard RNN based on SHAP; (B) Importance matrix of simulated data with TV-RNN based on SHAP.

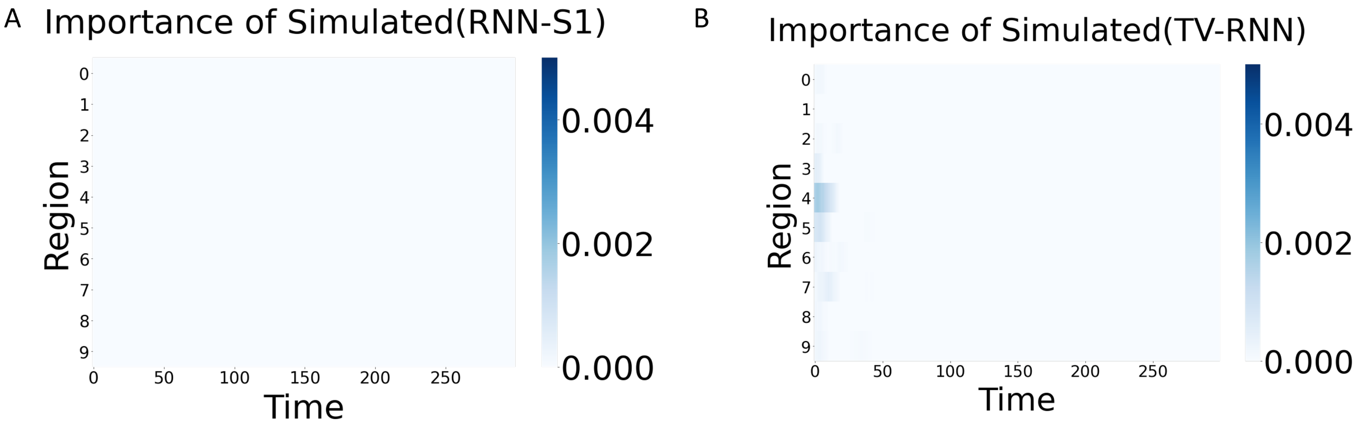

Fig. S17. (A) Importance matrix of simulated data with standard RNN based on Occlusion; (B) Importance matrix of simulated data with TV-RNN based on Occlusion.

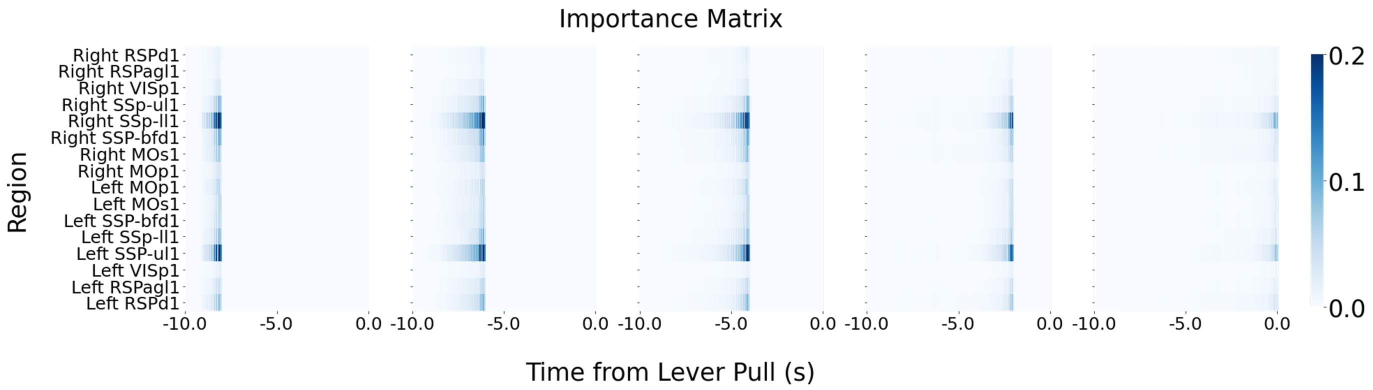

Fig. S18. Importance matrix of one example mouse (trained with trials combined). The importance is from the mean absolute value of SHAP across trials by using different temporal output, i.e., from 8 seconds before the behavior to 0 second before the behavior.
